# Mirtronic miR-4646-5p promotes gastric cancer metastasis by regulating ABHD16A and metabolite lysophosphatidylserines

**DOI:** 10.1038/s41418-021-00779-y

**Published:** 2021-04-19

**Authors:** Liping Yang, Yixuan Hou, Yan-e Du, Qiao Li, Fanlin Zhou, Yu Li, Huan Zeng, Ting Jin, Xueying Wan, Shengdong Guan, Rui Wang, Manran Liu

**Affiliations:** 1 Key Laboratory of Laboratory Medical Diagnostics designated by Chinese Ministry of Education, Chongqing Medical University, Chongqing, 400016 China; 2 Experimental Teaching Center of Basic Medicine Science, Chongqing Medical University, Chongqing, 400016 China; 3 Department of Laboratory Medicine, the Second Affiliated Hospital, Chongqing Medical University, Chongqing, 400010 China; 4 Department of Pathology, Chongqing University Cancer Hospital, Chongqing, 400030 China

**Keywords:** Metabolomics, Metastasis

## Abstract

The aberrant classical miRNAs are considered to play significant roles in tumor progression. However, it remains unclear for nonclassical miRNAs, a set of Drosha-independent miRNAs in the process of various biology. Here, we reveal that a nonclassical miR-4646-5p plays a pivotal role in gastric cancer (GC) metastasis. MiR-4646-5p, one of Drosha-independent mirtronic miRNA, is aberrant up-regulated in Drosha-low expressed GC and Drosha-knockdown gastric cancer cells. Mirtronic miR-4646-5p is a specific transcription splicing product of intron 3 of the host gene *Abhd16a* with the aid of SRSF2. The enhanced miR-4646-5p can stabilize HIF1A by targeting PHD3 to positive feedback regulate *Abhd16a* and miR-4646-5p itself expressions. ABHD16A, as an emerging phosphatidylserine-specific lipase, involves in lipid metabolism leading to lysophosphatidylserines (lyso-PSs) accumulation, which stimulates RhoA and downstream LIMK/cofilin cascade activity through GPR34/Gi subunit, thus causes metastasis of gastric cancer. In addition, miR-4646-5p/PHD3/HIF1A signaling can also up-regulate RhoA expression and synergistically promote gastric cancer cell invasion and metastasis. Our study provides new insights of nonclassical mirtronic miRNA on tumor progress and may serve as a new diagnostic biomarker for gastric cancer. MiR-4646-5p and its host gene *Abhd16a* mediated abnormal lipid metabolism may be a new target for clinical treatment of gastric cancer.

## Introduction

Gastric cancer (GC) is one of the most common lethal malignancies and the third leading cause of cancer mortality worldwide [1]. Although the incidence of gastric cancer has been declined in recent years [2], most of the cases still harbor a poor prognosis mainly due to high rate of recurrence and distant metastasis at advanced stages [3]. Therefore, it is necessary to explore the molecular mechanism underlying the progression and metastasis of GC and investigate mechanism-based therapeutic strategies for patients.

Drosha is a key enzyme for the generation of classical miRNA [4], and its aberrant expression causes anomalous processing of microRNAs, participating in carcinogenesis and cancer progression [5]. At present, inconsistent expression of Drosha has been observed in different human cancers [6]. Moreover, in gastric cancer, the relationship between Drosha and the risk of cancer remains disputed. Previous study of tissue microarrays (TMAs) has demonstrated a continuous reduction in cytoplasmic and nuclear Drosha during GC development [6]. However, a recent study has shown that mRNA expression level of Drosha was significantly upregulated in gastric tumoral tissues compared with marginal tissues, which was not related with clinical manifestations of the patients [7]. Another research showed that Drosha silence impedes the gastric tumor invasion [5]. Yet, the decreased Drosha was also observed in node-positive GC patients in comparison with node-negative GC patients and linked with dismal prognosis [8]. Based on these complicated statuses of Drosha in tumors, we carefully reanalyzed our previous TMAs data by bioinformatics, we found a few of GC patients with low Drosha expression do have high malignant characteristics and worse prognosis. Interestingly, we also found that some nonclassical miRNAs are abnormally elevated in Drosha-knockdown gastric cancer cells and Drosha-low expressed GC tissues. It raises an interesting question whether the abnormally elevated nonclassical miRNAs (Drosha-independent miRNAs) might be involved in GC malignant progression.

Currently, mirtron is the most common Drosha-independent miRNA. Mirtron is derived from the specific splicing of the intron of its host gene, which marries intron splicing with Drosha cleavage [9]. Due to its distinctive structural characteristics, a plethora of potential human mirtrons have been identified by computational analyses [10]. However, there has been little knowledge on its biological functions and biochemical regulation of mirtrons. Furthermore, few findings have underlined the specific expression profiles of mirtronic miRNAs in colorectal, stomach, and pancreatic cancer [11]. Yet the roles of specific mirtron in tumors remain unknown. Our previous study showed mirtronic miR-6778-5p strengthens gastric cancer stem cell stemness [12]. Here, we unveiled the functions of another aberrant mirtronic miR-4646-5p and its host gene Abhydrolase domain containing 16A *(Abhd16a)* in GC, which may shed light on gastric cancer malignancy and metastasis.

Metabolic reprogramming and aberrant activity of metabolic enzymes have been characterized as hallmarks of malignant tumors [13]. ABHD16A is a member of the α/β hydrolase domain-containing (ABHD) protein family, with acylglycerol lipase and phosphatidylserine lipase activities [14]. A study investigating the enzymatic characteristics of human ABHD16A in vitro revealed that ABHD16A is a MAG lipase with preference for medium-chain and long-chain fatty acids, especially long-chain unsaturated monoglycerides and 15-deoxy-Δ12,14-prostaglandin J2-2-glycerol ester (15d-PGJ2-G) [15]. Importantly, the interplay between ABHD16A and ABHD12 dynamically regulates immunomodulatory lysophosphatidylserines (lyso-PSs) and consequently affects the release of lipopolysaccharide-induced proinflammatory cytokines from macrophages [16]. In addition, ABHD16A may be an immune-balancing regulator that catalyzes the hydrolysis of prostaglandin-glycerol (PG-G) in neutrophils [17]. These researches suggest that ABHD16A, as an emerging lipase, impacts lipid metabolism and its metabolite levels. However, whether ABHD16A has a function in tumors, especially, ABHD16A-mediated lipid metabolism contributing to tumor development and metastasis remains largely unknown.

Lipid metabolites are bioactive lipids that normally act as signaling molecules modulating different cellular processes [18]. lyso-PS, derived from phosphatidylserine (PS) by the loss of a fatty acyl chain, has emerged as a new class of signaling molecule that modulates immune cell functions such as histamine release, T-cell proliferation and phagocytosis by signaling through G protein-coupled receptors (GPCRs) and Toll-like receptors [19]. So far, however, there has been little knowledge about the role of lyso-PS on tumor initiation and progress. Previous studies have revealed that lyso-PS stimulates the chemotactic migration of human glioma cell U87 or colorectal cancer cells through the GPR34 and PI3K/Akt pathways, suggesting that lyso-PS is associated with tumor invasion and metastasis [20]. A recent study reported that lyso-PS is a major component of lysophospholipids in colon cancer tissues and may be related to the development of colon cancer [21]. In addition, a significant increase in lyso-PS was detected in the ascites of gastric cancer patients, suggesting that it plays an important role in the malignant lesions of gastric cancer [22]. Thus, the effect of lyso-PS on gastric cancer metastasis and its molecular mechanism worth considerable critical attention.

The purpose of our study was to explore the effect of mirtronic miR-4646-5p and its host gene *Abhd16a* on lipid metabolism and gastric cancer metastasis. We found that Drosha low expression in some GC led to the enhanced mirtronic miR-4646-5p, which was spliced from the transcript of host gene *Abhd16a*. Elevated miR-4646-5p stabilized HIF1A through its target PHD3, which further positive feedback promoted *Abhd16a* transcription, miR-4646-5p itself upregulation, ABHD16A protein increase and accumulation of lyso-PS. The elevated lyso-PS contributed to activation of RhoA through GPR34/Gi, thus resulting in gastric cancer metastasis. In addition, miR-4646-5p/PHD3/HIF1A also upregulated RhoA expression. Thus miR-4646-5p/ABHD16A/lyso-PS stimulated activation of RhoA and miR-4646-5p/PHD3/HIF1A mediated RhoA expression synergistically promoted LIMK/cofilin signaling activity to fuel GC metastasis.

## Materials and methods

### Tissue specimens

GC tissue microarrays (TMAs) (ID: HStm-Ade178Sur-01, HStm-Ade180, HStm-Ade180Sur-01, HStm-Ade180Sur-02, OD-CT-DgStm, ST805, ST2091, ST2161, T011) including 374 normal adjacent tissue samples and 889 progressive gastric carcinoma were purchased from Xi’an Alena Biotech Co. Ltd. (Shanxi Province, China) and Shanghai Outdo Biotech Co. Ltd. (Shanghai, China), which were approved by the Medical Ethics Committee for the Use of Human or Animal Subjects of Taizhou Hospital, Zhejiang Province. Of these, 715 patients with complete clinical information were used for statistical analysis. None of the patients took nicotinamide prior to surgery.

The fresh gastric tumor tissues and their adjacent normal tissues used in this study were obtained from patients with gastric cancer without previous radiotherapy or chemotherapy at the First Affiliated Hospital of Chongqing Medical University. The investigation was approved by the ethics committee of Chongqing Medical University.

### Cell culture and reagents

The human gastric cancer MGC-803, SGC-7901 and BGC-823 cells were kindly donated by Prof. Yang Ke of the Beijing Institute of Cancer Research (Beijing, China). MGC-803 cells were cultured in DMEM medium with 10% FBS (Gibco, Australia), and SGC-7901 and BGC-823 cells were maintained in RPMI-1640 medium with 10% FBS (Gibco, Australia) at 37 °C in humidified atmosphere containing 5% CO_2_.

The reagents used in this study are as follows: 40 μM Proteasome inhibitor MG132 (Selleck); 1 mg Lysophosphatidylserine (LPS 18:0) (Sigma Aldrich); 100 ng/ml PTX (Sigma Aldrich); 10 μM Rho-kinase inhibitor Y-27632 (Cayman Chemical).

### Plasmid construction, inhibitors, and miRNA mimics

The shRNA specifically targeting miR-4646-5p was separately constructed by sub-cloning into the pLVX-puro vector (Clontech, USA) at BamHI and EcoRI sites using PCR. The expression constructions of miR-4646-5p, ABHD16A, HIF1A, and RhoA were reconstructed by inserting the sequences of miR-4646-5p, ABHD16A, HIF1A and RhoA cDNA into the pBABE-puro vector at BamHI and EcoRI sites, respectively. And the lentivirus expression vectors of shRNAs against Drosha, HIF1A, PHD3, ABHD16A, GPR34, SRSF2, and the control shRNA were purchased from GenePharma (Shanghai, China). Mimics, inhibitors and antagomir of miR-4646-5p were also purchased from GenePharma (Shanghai, China).

Plasmid construction used for splicing analysis of miR-4646/*Abhd16a* was established as described previously [12]. In brief, Mini-gene construct of miR-4646/ *Abhd16a* (exon 3–4) was amplified from human genomic DNA by RT-PCR with the Phusion High-Fidelity DNA Polymerase (ThermoFisher Scientific). PCR products were digested with BamHI and HindIII and inserted into pcDNA3.1 plasmid. 5′- and 3′-splice site mutations were made using Phusion High-Fidelity DNA Polymerase with primers containing splicing site mutations.

To generate wild type PHD3 (WT-PHD3) 3′-UTR-Luc and mutant PHD3 (Mut-PHD3) 3′-UTR-Luc reporters, the synthetic oligonucleotides (Invitrogen) that correspond to the wild-type or mutated binding sites of miR-4646-5p in the 3′-UTR of PHD3 were separately cloned into the pMIR-Reporter vector (Ambion, USA) at Spe1-HindIII sites. The binding sites were identified using the TargetScan database (MIT, www.targetscan.org). All the sequences described above are listed in Supplementary Table S1 and S2.

### RNA preparation and qRT-PCR

Total RNA was isolated using Trizol (Invitrogen, Carlsbad). PrimeScript RT Reagent Kit (TaKaRa, Dalian, China) was used for reverse transcription of the purified RNA. The cDNA was subjected to qRT-PCR with SYBR Premix Ex Taq™ II (TaKaRa, Dalian, China). Relative gene and miRNA expressions were detected using the 2^Ct (internal control) – Ct (gene)^. The primers used in this study are listed in Supplementary Table S3.

### Western blot analysis (WB analysis)

Gastric cancer cells were collected and lysed with RIPA buffer (containing protease inhibitors) to extract the total protein. Equal amounts of proteins were resolved via SDS–PAGE and transferred to PVDF membranes. The membranes were then blocked with 5% non-fat dry milk in TBS for 2 h and incubated overnight at 4 °C with the primary antibodies against the following proteins: anti-Drosha (1:1000; ab12286, Abcam), anti-ABHD16A(BAT5) (1:1000; ab185549, Abcam), anti-PHD3 (1:1000; ab30782, Abcam), anti-HIF1A (1:1000; ab51608, Abcam), anti-SRSF2(SC35) (1:1000, ab204916, Abcam), anti-Ubiquitin (1:1000; Abcam), anti-GPR34 (1:1000; ab134811, Abcam), anti-RhoA (1:500; ab187027, Abcam); anti-LIMK1 (1:1000; ab119084, Abcam), anti-p-LIMK1 (1:1000; ab38508, Abcam), anti-cofilin (1:1000; ab42824, Abcam), anti-p-cofilin (1:1000; ab12866, Abcam) and anti-β-Actin (1:1000; Bioshap). Next, the membranes were incubated in a secondary antibody for 1.5 h and Images were captured using Scion image software.

### Immunohistochemistry (IHC)

Immunohistochemical staining was performed as described previously [23]. Briefly, the deparaffinized tissue sections were heated for antigen retrieval, quenched for endogenous peroxidase activity and blocked with goat serum; the primary antibodies against Drosha (1:150; Abcam), anti-ABHD16A (BAT5) (1:200; GeneTex), anti-GPR34 (1:150; Abcam), anti-HIF1A (1:150; Abcam), anti-RhoA (1:150; Proteintech), anti-p-LIMK1 (1:150; Abcam), anti-p-cofilin (1:150; Abcam), and secondary antibody (1:100; ZSBIO, Beijing, China) were used. After staining with diaminobenzidine and hematoxylin, the images were captured.

Immunoreactivity was evaluated independently by two researchers who were blinded to patient outcome. Scoring criteria for staining intensity were as follows: 0 (negative), 1 (weak), 2 (moderate), 3 (strong). The staining extent score was as follows: 0 (<10%), 1 (11–25%), 2 (26–50%), 3 (51–75%), and 4 (76–100%). The final expression score, ranging from 0 to 12, was calculated as intensity scorexextent score. Total scores of 4 or lower were categorized as the low expression group, and the rest as the high expression group.

### Immunoprecipitation and cellular activated RhoA detection

Cells were seeded into six-well plates at a density of 1 × 10^5^ cells in 2 ml DMEM per well and transfected with constructor according to the designed experiments. After culturing for 16 h, cells were collected and lysed in NP-40 buffer. Anti-HIF1A agarose beads were used to pull down HIF1A. All experiments were performed at least three times.

RhoA activation was detected by analyzing GTP-bound RhoA utilizing the Rho-binding domain (RBD) from the effector protein Rhotekin as a probe in the active form of Rho-A pull-down assay [24]. The RhoA assay kit (Millipore, MA) was used to examine RhoA activation according to the manufacturer’s instructions.

### Chromatin immunoprecipitation (ChIP) assay and Luciferase reporter assay

ChIP assays were conducted with ChIP Kit (Thermo, MA, USA) using HIF1A and IgG antibodies. The primers used for amplifying potential binding site of HIF1A in the *Abhd16a* and *RhoA* were shown in Supplementary Table S4. The promoter of *Abhd16a* or *RhoA* with HIF1A binding motif (WT) or without HIF1A binding motif (MUT) was constructed into the pGL3 basic system (Promega). The pGL3-ABHD16A-promoter-WT or the pGL3-ABHD16A-promoter-Mut and the pGL3-RhoA-promoter-WT or the pGL3-RhoA-promoter-Mut were transfected into the MGC-803 cells together with the pCMV-Renilla control. Following the transfection for 30 h, the luciferase activity was detected. Transient transfection and luciferase assays were performed as described previously [12]. HEK-293, MGC-803 or SGC-7901 cells were co-transfected with miR-4646-5p mimics (GenePharma, Shanghai) and pMIR-PHD3 3′-UTR (wild-type or mutant) and pRL-TK control vector using lipofectamine 2000 (Invitrogen, Carlsbad). Renilla and firefly luciferase activities were measured with a Dual-Luciferase Reporter System (Promega, USA) after culture for 30 h.

### Cell invasion assay

Cell invasion were measured by a modified Boyden chamber assay as described previously [25]. In brief, MGC-803 (3 × 10^4^) or SGC-7901 (7 × 10^4^) cells in 200 μl serum-free medium were seeded into the wells of 8 μm-pore Boyden chambers (Millipore, Darmstadt, Germany) coated with Matrigel (1:7.5, Millipore, USA). Medium with FBS was added into the lower chamber. After incubation for the indicated time, the invaded cells on the opposite side of the filter were stained with crystal violet in methanol and counted under microscope. All the experiments were repeated in three times.

### Metabolite analysis

GC cells, including MGC-803 with ectopic miR-4646-5p or ectopic *Abhd16a*, and MGC-803 Drosha KD cells with shRNA against miR-4646-5p or shRNA against *Abhd16a*, and GC tissue samples were collected and flash-frozen in liquid nitrogen for extracting metabolites. Metabolomic analysis was performed using liquid chromatography mass spectroscopy (LC/MS-MS) as previously described [21, 26]. The raw LC-MS data were converted to mzXML format using ProteoWizard (version 3.0.19282), and further analyzed by XCMS. Metabolites were identified by accurate mass search and MS/MS spectral match using an in-house standard MS/MS library. And for the tissue sample, the concentrations of LPSs were calculated by obtaining the ratios between the LPS peaks and the IS (internal standards) peaks (1 mM of LPS species). The ratios of peak areas between the analytes and ISs were then used for quantifying the LPSs in samples with a calibration curve.

### In vivo metastasis assay

To evaluate the metastatic ability of GC cells in vivo, tail vein injection mouse model was performed. 4-week-old male BALB/c nude mice were separated randomly into 11 groups (*n* = 5 for each group). About 2 × 10^6^ MGC-803 cells transfected with constructor and/or treated with specific reagents according to the designed experiments were tail-vein injected into nude mice, respectively. Four weeks later, the nude mice were sacrificed. The lungs of nude mice were subjected to hematoxylin and eosin (H&E) staining for lung metastasis of GC cells. To further measure the metastases in liver, 1 × 10^6^ gastric cancer cells were injected into spleens of mice (*n* = 5 per group). After 6 weeks, the numbers of liver metastases were assessed by H&E staining. Ethics statement Animal experiments were permitted by the animal use committees of Chongqing Medical University. All animal experiments were conducted in accordance with an approved protocol and carried out according to the institutional animal welfare guidelines of the Chongqing Medical University.

### Statistical analysis

Statistical significance was determined using SPSS 19.0 software. The results are shown as means ± SD at least three independent determinations. Multiple groups were analyzed using ANOVA followed by the Student–Newman–Keuls multiple comparison test, and single comparison between two groups was analyzed using Student’s *t*-test. A value of *P* < 0.05 were considered statistically significant.

## Results

### 1. Reduced Drosha in gastric cancer causes up-regulation of Drosha-independent mirtronic miR-4646-5p, which is related to gastric cancer metastasis and poor prognosis

The controversial Drosha was reported in tumors [6]. By analysis of a dataset with GC recurrent and metastatic status from Oncomine database, we found GC patients with low Drosha (RNASEN) expression accounted for a large proportion of patients with recurrence (43.75%) (Supplementary Fig. S1A) and metastasis (45.16%) (Supplementary Fig. S1B). Moreover, Kaplan–Meier (KM) survival curves showed that in GC advanced stage (Stage 4, *n* = 148; N1 + 2 + 3, *n* = 422; M1, *n* = 56), Drosha low expressed patients had worse prognosis (Supplementary Fig. S1C–E). These findings indicated that there is decreased Drosha in some GC tissues, which is related with GC malignancy. To further investigated the relationship between Drosha low-expression and clinical characters, we reanalyzed the IHC score of Drosha proteins and clinical pathological parameters in Drosha reduced GC patients (IHC < 6) of TMAs. As shown in Supplementary Fig. S1F, 46.17% of Drosha low expressed patients (*n* = 163) were in stage III-IV, suggesting that lower Drosha expression is closely related to GC tumor progression (Supplementary Fig. S1F). These data demonstrate that there are a few of GC patients with reduced Drosha having high malignant characteristics and worse prognosis.

**Fig. 1:**
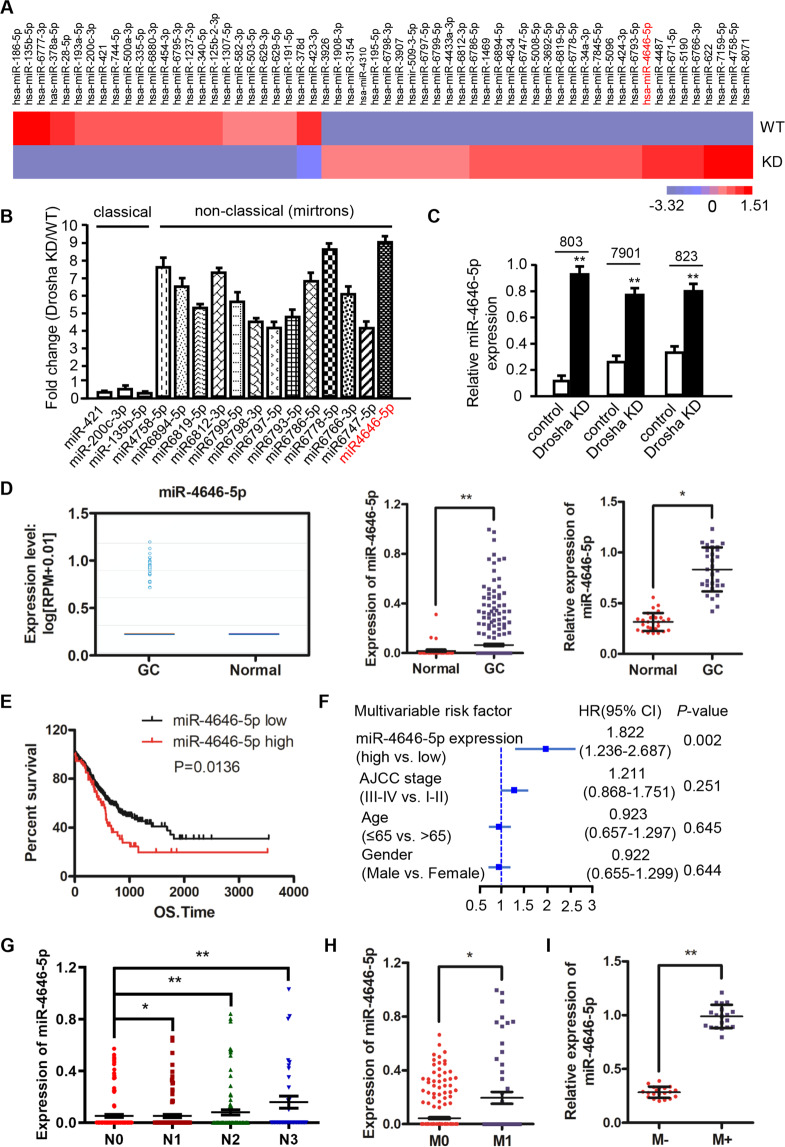
Aberrantly high-expressing Drosha-independent mirtronic miR-4646-5p was present in Drosha-knockdown GC cells, which are related to GC metastasis and poor prognosis. **A** The heatmap of down- or up-regulated miRNAs (top 30 miRNAs) identified by miRNA array in MGC-803 Drosha-knockdown (KD) cells. **B** The change of 3 classic miRNA and 13 of selected Drosha-independent mirtrons in microarray were validation by qRT-PCR. **C** qRT–PCR analysis of miR-4646-5p expression in Drosha WT and Drosha KD gastric cancer cells (**p < 0.01). **D** Expression of miR-4646-5p in gastric cancers from starBasev3.0 (left panel), or in normal and gastric tumor tissues from TCGA STAD data (middle panel), and the relative expression of miR-4646-5p in our cohort of clinical gastric cancer tissue (*n* = 30) and corresponding normal gastric tissue (*n* = 30) (right panel) (*p < 0.05, **p < 0.01). **E** Survival curve of gastric cancer patients with low or high miR-4646-5p expression (*p* = 0.0136) from TCGA STAD data. **F** Multivariable risk analyses of miR-4646-5p using TCGA STAD data. All the bars correspond to 95% confidence intervals. **G** Expression of miR-4646-5p in gastric cancer samples without lymph node metastases (N0) compared to samples with lymph node metastases (N1–N3). miRNA expression data and patient information were downloaded from UCSC Xena (https://xena.ucsc.edu/) (*p < 0.05, **p < 0.01). **H** TCGA STAD data to show miR-4646-5p levels in GC patients with distant metastases (M1) and non-distant metastases (M0) groups (*p < 0.05). (I) miR-4646-5p levels in GC tissues from the patients with metastases (M+, *n* = 20) and the age- and sex-matched patients without metastases (M−, *n* = 20) (**p < 0.01).

As Drosha is one of the key enzymes for classic miRNA production [4], we next investigated whether the decreased Drosha impacts on miRNA expressions in gastric cancer. Performing miRNA array analysis, we unveiled a widespread down-regulation of classic miRNAs in Drosha-knocked down (Drosha KD) MGC-803 cells compared with the wild type (Drosha WT) cells. More interestingly, some nonclassical miRNAs were abnormally elevated in the Drosha-knocked down gastric cancer cells (Fig. 1A). Nonclassical miRNAs (or called Drosha-independent miRNAs) were documented to be processed by alternative miRNA biogenesis pathways that bypass Drosha [27], including mirtrons (5′-tail mirtrons and 3′-tail mirtrons) and 5′-capped miRNAs (Supplementary Fig. S2A). Mirtron was the major Drosha-independent miRNA, originated from short hairpin intron alternative splicing of its host gene as previous reported [9]. Indeed, by analyzing the precursors and their host genes of the abnormally elevated miRNAs using UCSC database, we identified 13 potential Drosha-independent mirtrons in our miRNA profile (Supplementary Fig. S2B). To validate the microarray results, we further checked their expression changes of 13 potential mirtrons and 3 classical miRNAs (miR-421, miR-200c-3p, and miR-135b-5p) in MGC-803 Drosha KD cells by qRT-PCR, among which miR-4646-5p and miR-6778-5p were the highestly upregulated mirtrons (Fig. 1B). Given our previous observations that miR-6778-5p regulates gastric cancer stem cell stemness [12], we next focused on the effects of miR-4646-5p on the malignancy of gastric cancer, which was also significantly increased in other Drosha KD GC cells (Fig. 1C). These data suggest that Drosha knockdown or low-expression in gastric cancer cells is accompanied by abnormal elevation of nonclassical mirtrons, especially mirtronic miR-4646-5p.

To explore the relationship between the enhanced mirtronic miR-4646-5p and the malignancy of gastric cancer, we first detected miR-4646-5p levels in gastric cancer tissues. Using the data from starBASE database, we found that miR-4646-5p was highly expressed in gastric cancer patients (Fig. 1D, left panel), which was further confirmed by the TCGA STAD data (Fig. 1D, middle panel), and also verified in our cohort of GC patients tested by qRT-PCR (Fig. 1D, right panel). Next, we analyzed the relationship between miR-4646-5p expressions and GC patient outcomes. The survival curve showed that high-expressed miR-4646-5p was positively related to poor prognosis (*p* = 0.0136) (Fig. 1E). And multivariate Cox analysis further confirmed that high expression of miR-4646-5p was independently associated with reduced overall survival times (Fig. 1F). More importantly, miR-4646-5p was also significantly upregulated in GC patients with lymph node metastasis (Fig. 1G) and distant metastasis (Fig. 1H) compared with those without metastases by analysis of TCGA data, which is consistent with the findings acquired from our GC patient cohort with (M+) or without (M−) metastases by qRT-PCR (Fig. 1I). We therefore speculated that miR-4646-5p is a gastric cancer metastasis associated mirtron.

**Fig. 2:**
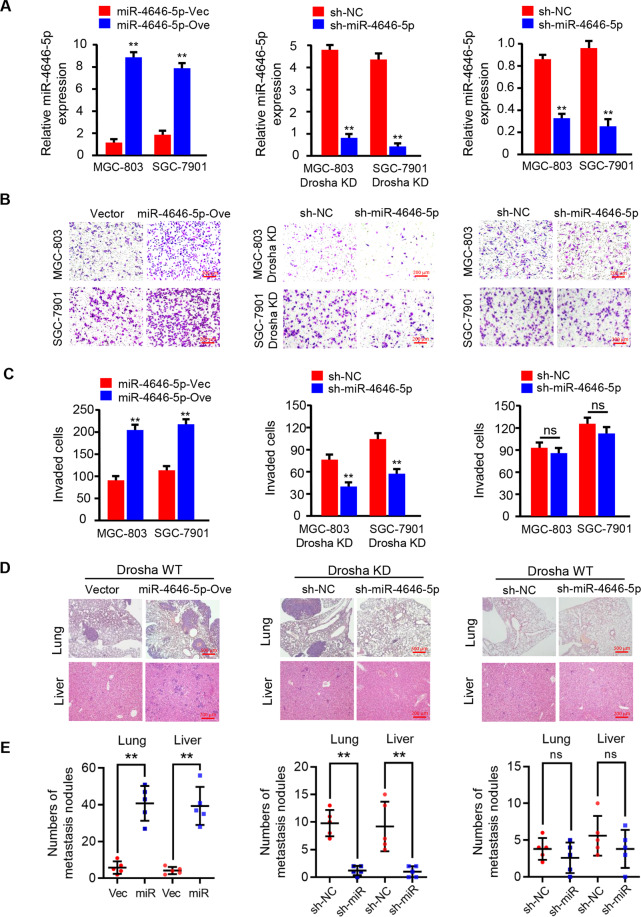
Mirtronic miR-4646-5p promotes cell invasion in vitro and metastasis in vivo in gastric cancer cells. **A** qRT-PCR to measure miR-4646-5p levels in GC cells: Drosha WT/miR-4646-5p and its control (left panel); Drosha KD/miR-4646-5p KD and its control (middle panel); Drosha WT/miR-4646-5p KD and its control (right panel) (Vec: vector; Ove: overexpression; **p < 0.01). **B**, **C** Transwell assay to assess cell invasion ability. The histograms show the average invaded cells each view (ns: no significance, **p < 0.01). **D**, **E** Effect of miR-4646-5p on GC metastasis in vivo by lung and liver metastasis model. Representative images and quantification of metastases in corresponding hematoxylin-eosin-stained lung sections (magnification, ×40; ns: no significance, **p < 0.01) and liver sections (magnification, ×100; ns: no significance, **p < 0.01).

To further confirm this hypothesis, we explored the relationship between miR-4646-5p expression and GC metastasis in vitro and in vivo. Using the cells with efficient transfection of ectopic miR-4646-5p into Drosha wild type-GC cells (Drosha WT/miR-4646-5p), or stably interfering miR-4646-5p expression in Drosha-knocked down GC cells (Drosha KD/miR-4646-5p KD), and the wild type GC cells (Drosha WT/miR-4646-5p KD) (Fig. 2A), we found that ectopic miR-4646-5p significantly increased GC cell invasion (e.g., Drosha WT/miR-4646-5p GC cells) (Fig. 2B, C, left panels), and loss of miR-4646-5p notably reduced tumor cell invasion abilities in Drosha-knocked down GC cells (e.g., Drosha KD/miR-4646-5p KD cells) (Fig. 2B, C, middle panels). However, the cell invasion ability had no much change between Drosha WT (Drosha WT/sh-NC) and Drosha WT with miR-4646-5p knocked down GC cells (Drosha WT/sh-miR-4646-5p) due to the low miR-4646-5p expression (Fig. 2B, C, right panels). Then, these GC cells were injected into mice to test their metastatic abilities to lung and liver tissue. Our data showed that more lung and liver metastases were acquired for the GC cells with Drosha WT/ectopic miR-4646-5p, fewer in the Drosha KD/miR-4646-5p KD group, and almost no significant different lung and liver metastases in mice injected with WT/sh-NC and Drosha WT/sh-miR-4646-5p GC cells (Fig. 2D, E, Supplementary Fig. S3A). Taken together, these data demonstrate that the reduced Drosha in gastric cancer is accompanied with an increase of Drosha-independent mirtrons, especially miR-4646-5p, which is associated with gastric cancer metastatic malignancy and poor prognosis.

### 2. Decreased Drosha and enhanced SRSF2 promote intron-specific splicing of the host gene *Abhd16a* to form mirtronic miR-4646-5p

**Fig. 3:**
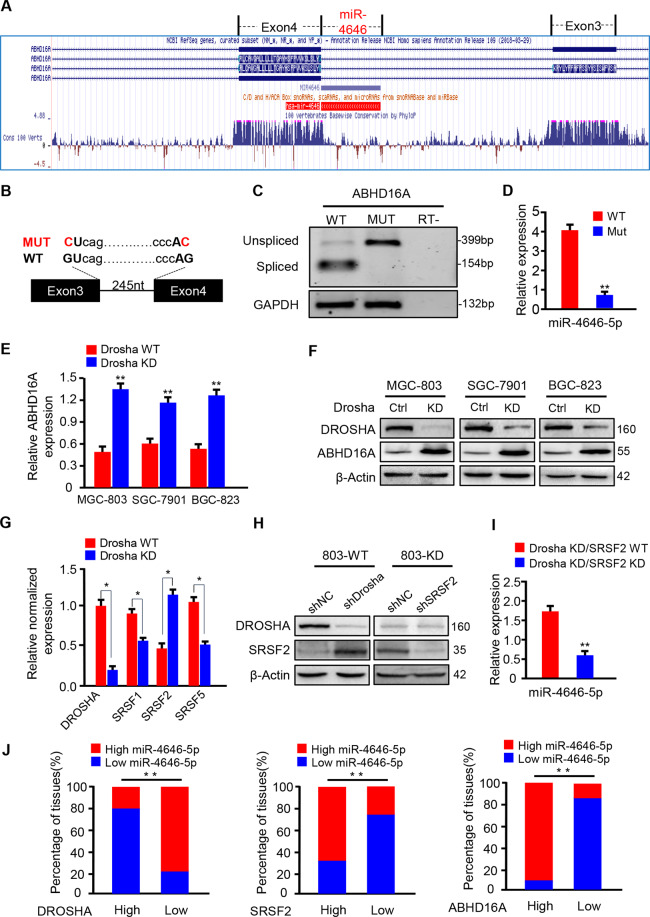
Drosha-independent 5′-tail mirtronic miR-4646-5p is a transcript splice derived from the intron-3 of *Abhd16a*. **A** Structure of 5′-tailed mirtronic miR-4646-5p using UCSC database (http://genome.ucsc.edu/). The fragment of pre-miR-4646-5p matched with the host gene *Abhd16a* is shown. Identification of Drosha-independent 5′-tail mirtron miR-4646-5p: **B** Minigene structure of miR-4646-5p. Boxes and lines represent exons and introns of *Abhd16a*, respectively. WT (wild type): splicing nucleotides in normal gene; MUT (mutant): minigene carrying the double mutant nucleotides in 5′-donor and 3′-acceptor, the mutant was performed from GU to CU, and AG to AC, respectively. **C** Splicing analysis of *Abhd16a* transcript. Wild type or splicing-deficient minigene transcript was transfected into MGC-803 cells, and mRNA products were tested by RT-PCR. Non-spliced form indicates transcript retaining mirtronic intron; spliced form represents the intron can be excised. RT-: control reaction, which was performed in the same condition without prior reverse transcription to exclude DNA contamination. GAPHD is loading control. **D** WT or Mutant minigene was transfected into MGC-803 cells, and miR-4646-5p level was detected by qRT–PCR. qRT–PCR (**E**) and western blotting (**F**) to detect ABHD16A expression in Drosha WT and Drosha KD gastric cancer cells (**p < 0.01). **G** qRT–PCR to detect the expression of splicing factors SRSF1, SRSF2, SRSF5 in Drosha-knocked down MGC-803 cells and control cells (*p < 0.05). **H** Protein levels of Drosha and SRSF2 were determined by western blotting in Drosha WT, Drosha KD, Drosha KD/SRSF2 KD and their control cells. **I** qRT-PCR to evaluate miR-4646-5p levels in Drosha KD/SRSF2 KD and its control cells (**p < 0.01). **J** Expression of miR-4646-5p, Drosha, SRSF2, and ABHD16A in 60 GC tissues. The investigated patients were classified into two groups (*n* = 30 per group) according to the median mRNA expression values of Drosha, SRSF2 and ABHD16A. And proportion of miR-4646-5p expression was shown in each group. (**p < 0.01).

To understand why mirtronic miR-4646-5p is upregulated in Drosha-low gastric cancer, we first explored the origin of miR-4646-5p. Using the UCSC database for sequence alignment, we found that the 5′ end of the precursor of miR-4646-5p is located at the junction of intron-3 and exon-4 of *Abhd16a* gene, indicating miR-4646-5p may be a 5′-tail mirtron, derived from intron-3 of *Abhd16a* gene (Fig. 3A). To further confirm miR-4646-5p being spliced from its host gene *Abhd16a*, we constructed a plasmid harboring minigenes of the intron-3 spanned by two coding exons (exon 3 and exon 4) of *Abhd16a*. Wild-type (WT) minigenes encompassed the natural introns, while mutant (MUT) variants of minigenes contained the intron with a mutation site of G residues at 5′ splice donor (GU changed to CU) and 3′ splice acceptor sites (AG changed to AC) (Fig. 3B). As shown in Fig. 3C, the intron was effectively spliced in mRNA processed from the plasmid with WT minigene in MGC-803 cells. In contrast, mRNA from the MUT variant retained the unspliced exon-intron-exon structure (Fig. 3C). In addition, the expression of miR-4646-5p was reduced in MGC-803 cells transfected with mutant minigene (MUT) compared to these transfected with wild type minigene (WT) (Fig. 3D). These data indicate that miR-4646-5p is a 5′-tail mirtron, which is formed by intron-3 specific splicing of its host gene *Abhd16a*. Interestingly, we also found that the mRNA and protein levels of ABHD16A were increased in Drosha knocked-down GC cells (Fig. 3E, F).

It has been reported that the altered expression of a particular splicing factor (SF) may affect the levels of mirtronic miRNAs [11], we then analyzed the expressions of splicing factors SRSF1, SRSF2, and SRSF5, which were identified by bioinformatics analysis of the splicing signals using the Human Splicing Finder online tool (Supplementary Fig. S3B). Like miR-4646-5p and *Abhd16a*, SRSF2 was significantly increased in MGC-803 Drosha KD cells (Fig. 3G, H). And knockdown of SRSF2 led to a decreased miR-4646-5p in Drosha-silenced GC cells (Fig. 3I), indicating high level of SRSF2 facilitates splicing of miR-4646-5p. Furthermore, by analyzing potential splice motifs of splicing factors, we identified only the specific splicing targets of SRSF2 located in the junctions of intron-3 and exon-3/4 of *Abhd16a* (Supplementary Fig. S3B), implying involvement of SRSF2 in mirtronic miR-4646-5p processing. To evaluate their clinical relevance of Drosha, SRSF2, ABHD16A and miR-4646-5p, we tested their expression levels in 60 samples of gastric cancer patients. As expected, the expression of miR-4646-5p is negatively correlated with Drosha, and positively correlated with SRSF2 and ABHD16A (Fig. 3J), which was also confirmed with TCGA data from UCSC Xena (Supplementary Fig. S3C). Taken together, these data demonstrate that high level of splicing factor SRSF2 in Drosha decreased GC cells promotes the intron-3 specific splicing of *Abhd16a*, leading to an upregulated mirtronic miR-4646-5p in GC.

### 3. miR-4646-5p targets PHD3 to stabilize HIF1A to feedback upregulate the host gene *Abhd16a* expression

**Fig. 4:**
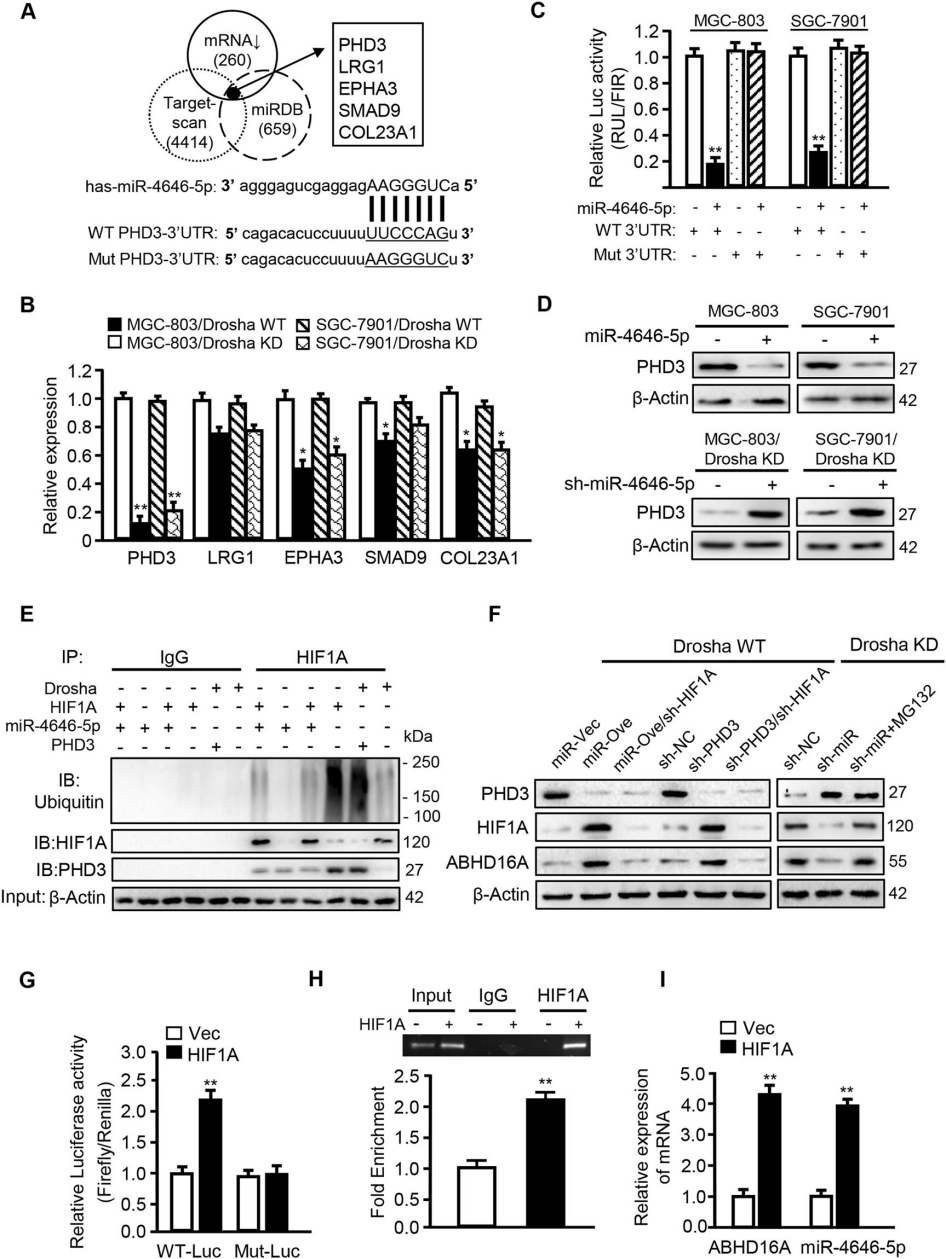
miR-4646-5p stabilizes HIF1A through its target gene *PHD3*, which feedback promotes the expression of host gene *Abhd16a*. *PHD3* is the target gene of miR-4646-5p (*p < 0.05, **p < 0.01). **A** The putative targets of miR-4646-5p were predicted by miRDB (659 genes) and Targetscan (4414 genes), and intersected with the low-expressed mRNA (260 genes) in Drosha-knockdown GC chips. The 3′-UTR binding site of the target *PHD3* is shown (WT and MUT). **B** The expression of *PHD3*, *LRG1*, *EPHA3*, *SMAD9* and *COL23A1* were detected by qRT-PCR in Drosha KD GC cells. **C** The wild-type PHD3 3′-UTR (WT) reporter or miR-4646-5p binding sites mutant (MUT) reporter was transfected into MGC-803 or SGC-7901 cells, the inhibition effect of miR-4646-5p on luciferase activities was detected. **D** PHD3 protein levels were determined by western blot analysis in MGC-803 and SGC-7901 cells transfected with ectopic miR-4646-5p or a control vector and in Drosha-knockdown MGC-803 and SGC-7901 or Drosha and miR-4646-5p double knockdown cells. Knockdown of *PHD3* inhibits HIF1A protein ubiquitination. **E** Immunoprecipitation to detect ubiquitination level of HIF1A. The ubiquitination of HIF1A was shown no difference when HIF1A was knockdown in MGC-803 Drosha KD cells, to be increased when miR-4646-5p was knockdown in MGC-803 Drosha KD cells, and be decreased when *PHD3* was knockdown in Drosha WT cells. **F** Western blot analysis was performed to determine PHD3, HIF1A and ABHD16A protein levels in the indicated gastric cancer cells. Drosha and miR-4646-5p doubly knocked down cells were treated with MG132 for 6 h. HIF1A as a transcription factor promotes *Abhd16a* expression. **G** Luciferase assay to show HIF1A regulating transcriptional activity of *Abhd16a* in MGC-803 cells (**p < 0.01). **H** Chromatin immunoprecipitation assay was conducted using extracts of MGC-803 transfected with ectopic *HIF1A* or control vector. IgG was used as negative controls, respectively (**p < 0.01). **I** qRT–PCR was used to determine *Abhd16a* and miR-4646-5p expression in MGC-803 cells transfected with ectopic HIF1A or control vector (**p < 0.01).

To identify the direct target of mirtronic miR-4646-5p, the potential target genes were predicted by miRDB and TargetScan, and then compared with the down-regulated genes in our mRNA profiles of Drosha knocked down MGC-803 cells (Fig. 4A). We identified five candidate genes (PHD3, LRG1, EPHA3, SMAD9, and COL23A1) (Fig. 4A), and PHD3 was the mostly downregulated one in Drosha-knockdown GC cells (Fig. 4B). To prove PHD3 is a positive target of miR-4646-5p, ectopic miR-4646-5p was transfected into Drosha WT GC cells. PHD3 expression was significantly reduced in the Drosha WT GC cells with ectopic miR-4646-5p (Drosha WT/miR-4646-5p), and increased in Drosha and miR-4646-5p doubly knocked down GC cells (Drosha KD/miR-4646-5p KD) compared to other candidate genes (Supplementary Fig. S4A). Moreover, miR-4646-5p could inhibit *PHD3* 3′-UTR luciferase activity, whereas mutation of the binding-site canceled the inhibitory effects of miR-4646-5p on *PHD3* 3′-UTR luciferase activity (Fig. 4C and Supplementary Fig. S4B). Furthermore, overexpression of miR-4646-5p in parental GC cells reduced PHD3 protein levels (Fig. 4D, upper panel), and knockdown of miR-4646-5p in Drosha-silenced GC cells increased PHD3 proteins (Fig. 4D, down panel), suggesting that *PHD3* is a direct target gene of miR-4646-5p and down-regulated in Drosha KD cells.

Given PHD3 is one of the key factors for HIF1A ubiquitination degradation [28], we wondered whether miR-4646-5p targeting PHD3 could change HIF1A ubiquitination levels. As expected, miR-4646-5p knockdown in Drosha-silenced GC cells facilitated the ubiquitination process of HIF1A, whereas *PHD3* knockdown in Drosha wild type GC cells reduced HIF1A ubiquitination levels (Fig. 4E). Correspondingly, HIF1A expression was up-regulated in Drosha WT MGC-803 with ectopic miR-4646-5p (MGC-803 Drosha WT/ miR-4646-5p) cells or *PHD3* silenced cells, and down-regulated in Drosha and miR-4646-5p double knockdown GC cells (MGC-803 Drosha KD/miR-4646-5p KD); and proteasome inhibitor MGC132 treatment, which inhibited ubiquitination process, partially restored the expression of HIF1A (Fig. 4F). Therefore, miR-4646-5p could mitigate ubiquitination of HIF1A by inhibiting the target gene *PHD3*, thus lead to an enhanced HIF1A in Drosha-knockdown gastric tumor cells.

Interestingly, the expression of ABHD16A had the same trend as HIF1A done in these GC cells. Moreover, once *HIF1A* was knocked down in Drosha WT MGC-803 with ectopic miR-4646-5p cells or *PHD3* silenced cells, the expression of ABHD16A was reduced (Fig. 4F, left panel), indicating a potential regulation of ABHD16A by HIF1A. To understand the intrinsic correlation between ABHD16A and HIF1A, we predicted that HIF1A is a potential transcription factor for *Abhd16a* using the JASPAR database (http://jaspar.genereg.net/) (Supplementary Fig. S4C). Indeed, as detected by Luciferase assay, HIF1A could regulate *Abhd16a* transcriptional activity (Fig. 4G). CHIP assays also revealed HIF1A enrichment on the *Abhd16a* promoter (Fig. 4H), confirming that HIF1A is a transcription factor of *Abhd16a*. In addition, overexpression of HIF1A upregulated expressions of the host gene *Abhd16a* and miR-4646-5p itself in MGC-803 cells (Fig. 4I). Thus, these data reveal a positive feedback regulation of miR-4646-5p/PHD3/HIF1A/ABHD16A in Drosha-decreased gastric tumor cells, that is, miR-4646-5p stabilizes HIF1A by targeting *PHD3* and feedback promotes expression of the host gene *Abhd16a* and miR-4646-5p itself.

### 4. ABHD16A, as a novel phosphatidylserine-specific lipase, involves in lipid metabolism-caused lyso-PS accumulation to promote gastric cancer metastasis

**Fig. 5:**
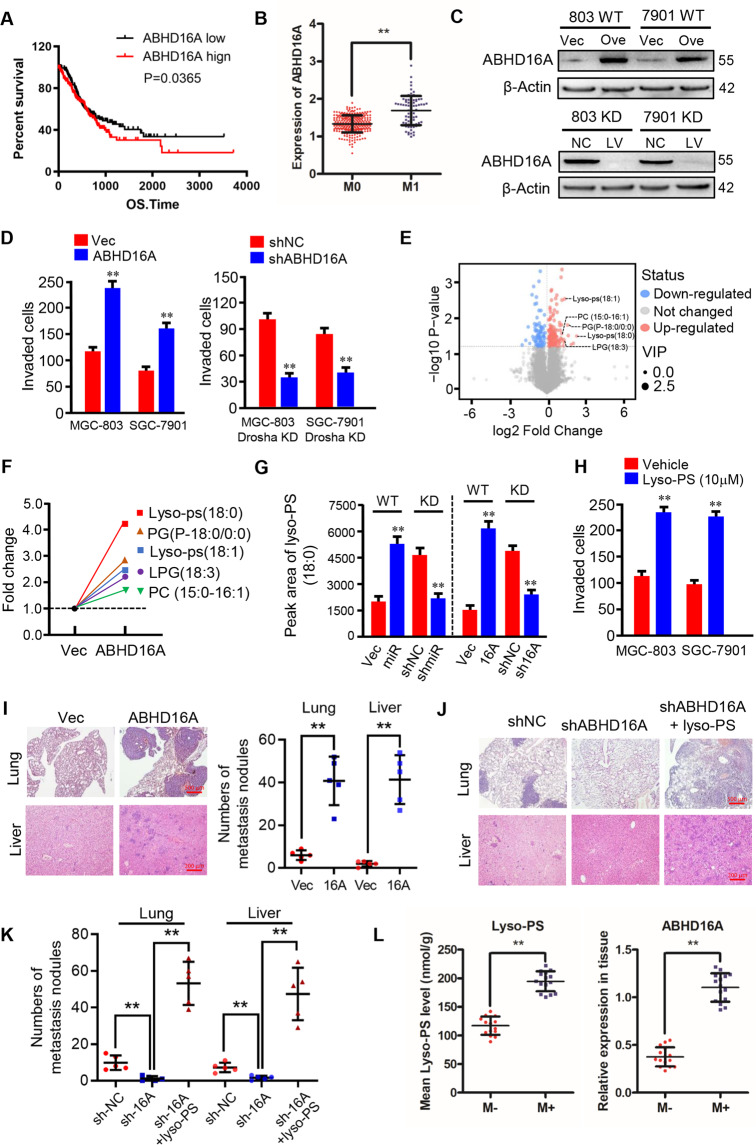
ABHD16A, as a novel phosphatidylserine-specific lipase, participated in lipid metabolism, which causes lyso-PS accumulation to promote gastric cancer metastasis. **A** Survival curve of gastric cancer patients with low or high ABHD16A expression (*p* = 0.0365). ABHD16A expression data and patient information were downloaded from TCGA database. **B** Using database from UCSC Xena, ABHD16A levels in gastric cancer patients with distant metastases (M1) and non-distant metastases (M0) were shown (**p < 0.01). **C** Western blot analysis to determine ABHD16A levels in MGC-803 and SGC-7901 cells transfected with ectopic *Abhd16a* or control vector (Upper panel), and in Drosha and *Abhd16a* doubly knocked down MGC-803 and SGC-7901 cells and their control cells (Down panel). **D** Transwell assay to test cell invasion of MGC-803 and SGC-7901 cells transfected with ectopic *Abhd16a* or control vector (left panel), or Drosha and *Abhd16a* doubly knocked down MGC-803 and SGC-7901 cells (right panel). The histograms show the average invaded cells each view (**p < 0.01). Metabolites were identified using LC/MS-MS as described in methods. **E** Volcano plot showing differentially changed metabolites in MGC-803 transfected with ectopic *Abhd16a* or control. **F** Fold changes of lipid classes (top 5 metabolites) between MGC-803 cells transfected with ectopic *Abhd16a* and control vector. **G** The concentration of lyso-PS (18:0) in the indicated engineered MGC-803 cells. The peaks area of lyso-PS (18:0) extracted by software LipidSearch 4.0 (Thermo Fisher Scientific, CA, USA) represents the concentration of lyso-PS (18:0) (**p < 0.01). **H** Transwell assay to test cell invasion of MGC-803 and SGC-7901 cells treated with or without lyso-PS (10 μM) (**p < 0.01). The effect of ABHD16A and lyso-PS on GC metastasis in vivo using lung and liver metastasis model. **I **Ectopic *Abhd16a* promoted GC lung and liver metastasis. **J**, **K** Loss of ABHD16A decreased GC lung and liver metastasis, and could be restored by exogenous addition of lyso-PS. Representative images of lung and liver, and quantification of metastases in corresponding hematoxylin-eosin-stained lung sections (magnification, ×40; **p < 0.01) and liver sections (magnification, ×100; **p < 0.01) were shown. **L** Intracellular lyso-PS concentration of GC tissues with metastases (M + , *n* = 15) or without metastases (M−, *n* = 15) were determined by LC/MS-MS. ABHD16A levels in the corresponding GC tissues were evaluated by qRT-PCR (**p < 0.01).

To understand the role of ABHD16A in tumor metastasis and prognosis of GC, 382 cases of gastric cancer from TCGA data were assessed. Like miR-4646-5p, high expression of ABHD16A in GC correlated with poor prognosis (Fig. 5A). Additionally, ABHD16A levels were higher in tumor with distant metastasis (M1) than tumor in site (M0) by analysis of STAD data from UCSC Xena (Fig. 5B). To further validate the role of ABHD16A in promoting gastric cancer metastasis, we generated the engineered gastric cells including GC cells with ectopic *Abhd16a* (Drosha WT/ABHD16A), and *Abhd16a* and Drosha doubly knocked down cells (Drosha KD/ABHD16A KD) (Fig. 5C, Supplementary Fig. S5A). As expected, restoration of ABHD16A in Drosha WT gastric cancer cells markedly increased tumor cell invasion potentials, while silencing *Abhd16a* in Drosha-knockdown GC cells (Drosha KD/ABHD16A KD) notably decreased their cell invasion abilities (Fig. 5D, Supplementary Fig. S5B), suggesting that ABHD16A plays an essential role for GC metastasis.

As ABHD16A is a novel phosphatidylserine-specific lipase which involves in lipid metabolism [19], we asked whether the effect of ABHD16A on gastric cancer metastasis was caused by altered lipid metabolites. Thus, metabolite analysis was performed using LC/MS-MS. Volcano plots showed the significant changes of lipid metabolites in ABHD16A overexpressing MGC-803 cells (MGC-803 Drosha WT/ABHD16A) in comparison with control MGC-803 cells (MGC-803 Drosha WT) (Fig. 5E). Among these metabolites, lysophosphatidylserines (lyso-PSs) (18:0, 18:1) were significantly increased in ABHD16A overexpressing GC cells (Fig. 5F). To confirm that the enhanced lyso-PS was induced by miR-4646-5p/ABHD16A axis, we next investigated the lyso-PS concentration in Drosha wild type GC cells with ectopic miR-4646-5p or ectopic *Abhd16a* (GC/Drosha/miR-4646-5p, and GC/Drosha/ABHD16A), or in Drosha-silenced GC cells with shRNA against miR-4646-5p or shRNA against *Abhd16a* (GC/Drosha KD/sh-miR-4646-5p, and GC/Drosha KD/sh-ABHD16A). As expected, the high level of lyso-PS (18:0) was detected in ectopic miR-4646-5p or ABHD16A expressing gastric cancer cells (GC/Drosha WT/miR4646-5p, and GC/Drosha WT/ABHD16A), loss of miR-4646-5p or ABHD16A in Drosha-silenced GC cells (GC/Drosha KD/sh-miR4646-5p, and GC/Drosha KD/sh-ABHD16A) led to decrease of lyso-PS (18:0) (Fig. 5G). Thus, these results confirm a crucial role of miR-4646-5p/ABHD16A axis to lyso-PS accumulation.

To determine whether the effects of miR-4646-5p and ABHD16A on GC invasion and metastasis were specifically due to lyso-PS accumulation, exogenous lyso-PS was added to growth medium and GC cell invasion was evaluated. As expected, lyso-PS treatment increased GC cell invasion (Fig. 5H, and Supplementary Fig. S5C). Furthermore, more lung and liver metastases were observed in mice injected with Drosha WT/ABHD16A overexpressing GC cell group compared with the Drosha WT GC cell group (Fig. 5I, Supplementary Fig. S5D left panel), whereas knockdown of *Abhd16a* in Drosha-knockdown GC cells significantly reduced metastases in mice lung and liver, and administration of lyso-PS for mice injected with Drosha KD/ABHD16A KD cells could partially restore tumor cell metastatic ability to lung and liver (Fig. 5J,K, Supplementary Fig. S5D right panel). To expand these findings in clinic, we detected lyso-PS and ABHD16A levels in gastric tumor tissues with or without metastases. Low level of lyso-PS (18:0) was detected in GC tissues without metastases (M−) which had low ABHD16A expressions; and high concentration of lyso-PS (18:0) was in GC tissues with metastases (M+) companied with enhanced ABHD16A (Fig. 5L). Taken together, these data demonstrate that ABHD16A, as a novel phosphatidylserine-specific lipase, plays an essential role to fuel gastric cancer metastasis via regulating lyso-PS accumulation in lipid metabolism.

### 5. HIF1A-mediated upregulation of RhoA and Lyso-PS/GPR34 stimulated activation of RhoA synergistically trigger LIMK/cofilin signaling to promote GC metastasis

To investigate the underlying mechanism of lyso-PS on GC metastasis, RNA sequencing was carried out. Analysis of mRNA information acquired from lyso-PS treated or non-treated MGC-803 cells by bioinformatics, several signaling pathways including GPCR ligand binding, G alpha (i) signaling, Rho protein transduction signaling and cell migration/invasion signaling were enriched under lyso-PS treatment (Supplementary Fig. S5E), implicating lyso-PS-GPCR-Gα(i)-Rho axis is the target downstream signaling of lyso-PS. GPCRs (e.g., GPR34, P2Y10, and GPR174) were reported to be the potential receptor of lyso-PS in human tissues [29]. Among them, only GPR34 was detected to be highly expressed in MGC-803, SGC-7901 and BGC-823 cells (Supplementary Fig. S5F). GPR34 can couple with members of Gi/Go-protein family [30]. As a promising molecular couple of Gi/Go-protein, activation of RhoA was thus tested under miR-4646-5p/ABHD16A-mediated lyso-PS accumulation.

**Fig. 6:**
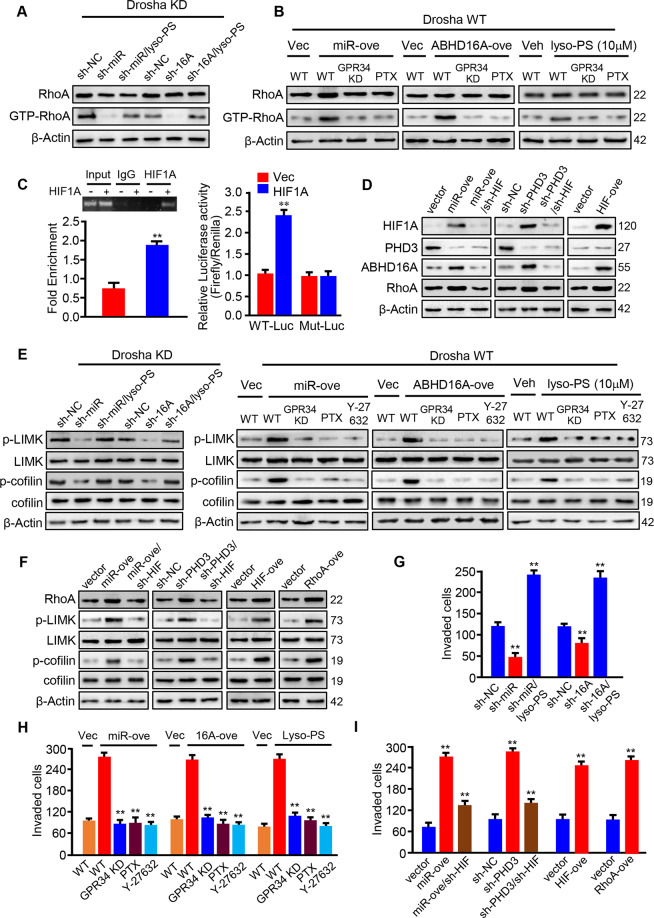
Lyso-PS activates RhoA, cooperates with HIF1A to regulate RhoA expression, promotes invasion of gastric cancer cell. Pull-down assays and western blotting to examine the activated RhoA (GTP-RhoA). **A** Silencing miR-4646-5p or *Abhd16a* in Drosha decreased GC cells reduced the activated RhoA, then be restored by lyso-PS (10 μM) administration. **B** miR-4646-5p and ABHD16A overexpression in gastric cancer cells or exogenous lyso-PS (10 μM) were shown to activate RhoA activity in GC cells with endogenous GPR34, but not in GPR34-silenced or PTX (inhibitor of Gα_i_, 100 ng/ml, 4 h) treated GC cells. **C** Chromatin immunoprecipitation assay was conducted using extracts of MGC-803 transfected with ectopic HIF1A or control vector. IgG was used as negative controls (**p < 0.01) (left panel). Luciferase assay to show HIF1A regulating transcriptional activity of RhoA in MGC-803 cells (**p < 0.01) (right panel). **D** Western blotting to determine HIF1A, PHD3, ABHD16A, and RhoA protein levels in MGC-803 cells with ectopic miR-4646-5p, ectopic *HIF1A* or silenced *PHD3* and the controls, or HIF1A reduced MGC-803 cells with ectopic miR-4646-5p or silenced *PHD3*. **E**, **F** Western blotting to detect p-LIMK and LIMK, p-cofilin and cofilin levels in the above GC cells. **G**–**I** Transwell assay to test cell invasion ability of GC cells mentioned in **E** and **F**. The histograms show the average invaded cells each view (**p < 0.01).

Using RBD from the effector protein Rhotekin as a probe to specifically pull down the active forms of RhoA, we detected high level of activated RhoA (GTP-bound RhoA) in Drosha knockdown GC cells (Drosha KD/shNC), which was then reduced when silencing miR-4646-5p or ABHD16A in Drosha decreased GC cells (Drosha KD/sh miR-4646-5p or Drosha KD/sh ABHD16A); and importantly, the reduction of activated RhoA could be rescued by lyso-PS administration (Drosha KD/sh miR-4646-5p/lyso-PS, or Drosha KD/sh ABHD16A/lyso-PS) (Fig. 6A). Furthermore, ectopic miR-4646-5p or *Abhd16a* in Drosha WT MGC-803 cells, or administrating Drosha WT MGC-803 cells with lyso-PS treatment significantly increased the activated RhoA levels (Fig. 6B); however, knockdown of GPR34, the potential receptor of lyso-PS in GC cells, neither ectopic miR-4646-5p/*Abhd16a* nor exogenous supplement of lyso-PS could increase RhoA activity (Fig. 6B); in addition, inhibition of Gi activity by pertussis toxin (PTX, Gi inhibitor, 100 ng/ml) in GC cells canceled the role of ectopic miR-4646-5p and *Abhd16a* or lyso-PS administration to stimulate the activation of RhoA in Drosha wild type GC cells (Fig. 6B), indicating miR-4646-5p/ABHD16A-mediated lyso-PS causes RhoA activity though receptor GPR34/Gi. On the other hand, HIF1A is a potential regulator of RhoA as previous reported [31]. Using luciferase reporter assay and ChIP assay, we verified that HIF1A could directly bind to the RhoA promoter to regulate RhoA transcription (Fig. 6C). Next, we wondered whether miR-4646-5p/PHD3 axis could impact HIF1A-mediated RhoA expression. Indeed, ectopic miR-4646-5p and HIF1A, or silencing PHD3 up-regulated RhoA expression in Drosha wild type MGC-803 cells, while silencing HIF1A again in Drosha wild type MGC-803 with ectopic miR-4646-5p (MGC803/miRNA4646-5p/shHIF1A) or shRNA against PHD3 (MGC803/shPHD3/shHIF1A) dramatically decreased RhoA protein levels (Fig. 6D), revealing that miR-4646-5p/PHD3/HIF1A axis involves in regulating RhoA expression. Taken together, miR-4646-5p/PHD3/HIF1A axis upregulates *RhoA* transcription and the RhoA activation can be triggered by miR-4646-5p/ABHD16A-mediated lipid metabolite lyso-PS accumulation in gastric cancer cells.

Next, we asked how the enhanced RhoA and activation of RhoA contribute to GC metastatic malignancy. RhoA was reported to impact activity of LIMK/cofilin cascade, an important signaling in tumor metastasis, by phosphorylation process [32]. Thus, the activated RhoA/LIMK/cofilin signaling impacting on GC cell invasion ability was evaluated. As expected, activated RhoA could promote phosphorylation of LIMK and cofilin in Drosha reduced GC cells. Namely, silence of miR-4646-5p or ABHD16A in Drosha decreased GC cells (Drosha KD/miR-4646-5p KD or Drosha KD/ABHD16A KD) reduced phosphorylated LIMK and phosphorylated cofilin levels, which could be restored by lyso-PS administration (Fig. 6E, left panel). Ectopic miR-4646-5p or *Abhd16a* and lyso-PS treatment notably increased phosphorylation of LIMK and cofilin in parent MGC-803 cells; however, silencing GPR34, or inhibiting Gi activity by PTX (100 ng/ml), or inhibiting activation of RhoA using Y-27632 (RhoA inhibitor, 10 μM) markedly reduced phosphorylation of LIMK and cofilin (Fig. 6E, right panel). These data suggest that miR-4646-5p/ABHD16A-mediated lyso-PS accumulation leads to RhoA activation, thus phosphorylating LIMK and cofilin in Drosha-decreased GC cells. On the other hand, miR-4646-4p/PHD3/HIF1A axis could upregulate RhoA expression to promote LIMK and cofilin phosphorylation in Drosha decreased GC cells. As shown, ectopic miR-4646-5p, *HIF1A* and *RhoA*, or silencing *PHD3* notably increased p-LIMK and p-cofilin levels in parent GC cells, while depletion of HIF1A again in the parent GC cells with ectopic miR-4646-5p or shRNA against *PHD3* reduced p-LIMK and p-cofilin proteins (Fig. 6F). Correspondingly, GC cell invasion had a similar change in accompany with the activation or expression of RhoA and the phosphorylation of LIMK/cofilin (Fig. 6G–I; Supplementary Figs. S6A, B, and S7A).

**Fig. 7:**
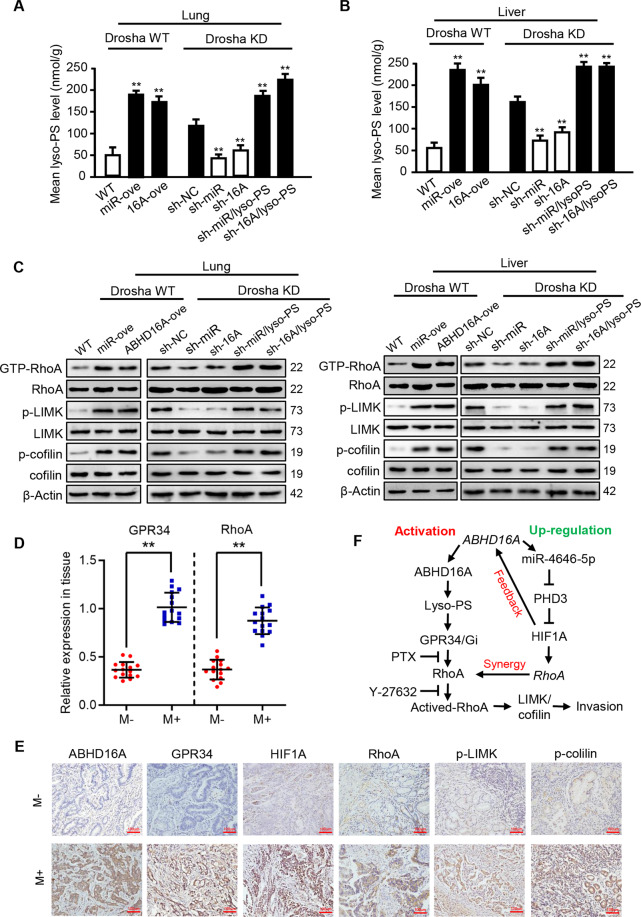
miR-4646-5p/ABHD16A/Lyso-PS stimulated activation of RhoA trigger LIMK/cofilin signaling to promote GC metastasis in vivo and clinic. **A, B** Lyso-PS concentration of the mice lung and liver metastasis tissues in subgroups were determined by LC/MS-MS (**p < 0.01). **C** The change of activated RhoA and RhoA, p-LIMK and LIMK, p-cofilin and cofilin in mice lung and liver metastasis tissues by Western blot. **D** The relative GPR34 and RhoA levels in gastric tumor tissues from the patients with metastases (M+, *n* = 20) were compared with the age- and sex-matched patients without metastases (M-, *n* = 20). **E** Immunohistochemical staining to detect ABHD16A, RhoA, HIF1A, GPR34, p-LIMK, and p-cofilin levels in metastatic GC tumors or in non- metastatic tumor (magnification, ×200; Scale bar, 100 µm). **F** Summary chart of ABHD16A regulating and activating RhoA and its downstream signaling. RhoA-mediated GC metastasis closely relates with miR-4646-5p/PHD3/HIF1A mediated upregulation of RhoA and lyso-PS/GPR34 depended activation of RhoA in GC cells.

To expand our findings of miR-4646-5p/ABHD16A-mediated lyso-PS accumulation and its downstream signaling activation on GC metastasis in vivo, we further assessed the levels of lyso-PS and the activated RhoA/LIMK/cofilin signaling in lung and liver metastases of mice. As shown in Fig. 7A, B, miR-4646-5p or ABHD16A overexpression in Drosha wild type GC cells led to a significant increase of lyso-PS in lung and liver metastases. Conversely, knockdown of miR-4646-5p or *Abhd16a* in Drosha silenced GC cells notably attenuated the lyso-PS concentration in lung and liver metastases, while this reduction could be rescued by exogenous supplementation of lyso-PS (Fig. 7A, B). Likewise, the activated RhoA and phosphorylated LIMK and cofilin had a similar change in company with the lyso-PS levels in lung and liver metastases (Fig. 7C). Meanwhile, in the lung and liver metastases, the RhoA protein was upregulated in GC cells with ectopic miR-4646-5p (Drosha WT/miR-4646-5p), while declined in Drosha and miR-4646-5p doubly knocked down GC cells (Drosha KD/miR-4646-5p KD) (Fig. 7C). Hence, miR-4646-5p/PHD3/HIF1A-mediated upregulation of RhoA and miR-4646-5p/ABHD16A/lyso-PS stimulated activation of RhoA synergistically trigger LIMK/cofilin signaling to promote GC metastasis in vitro and in vivo.

**Fig. 8:**
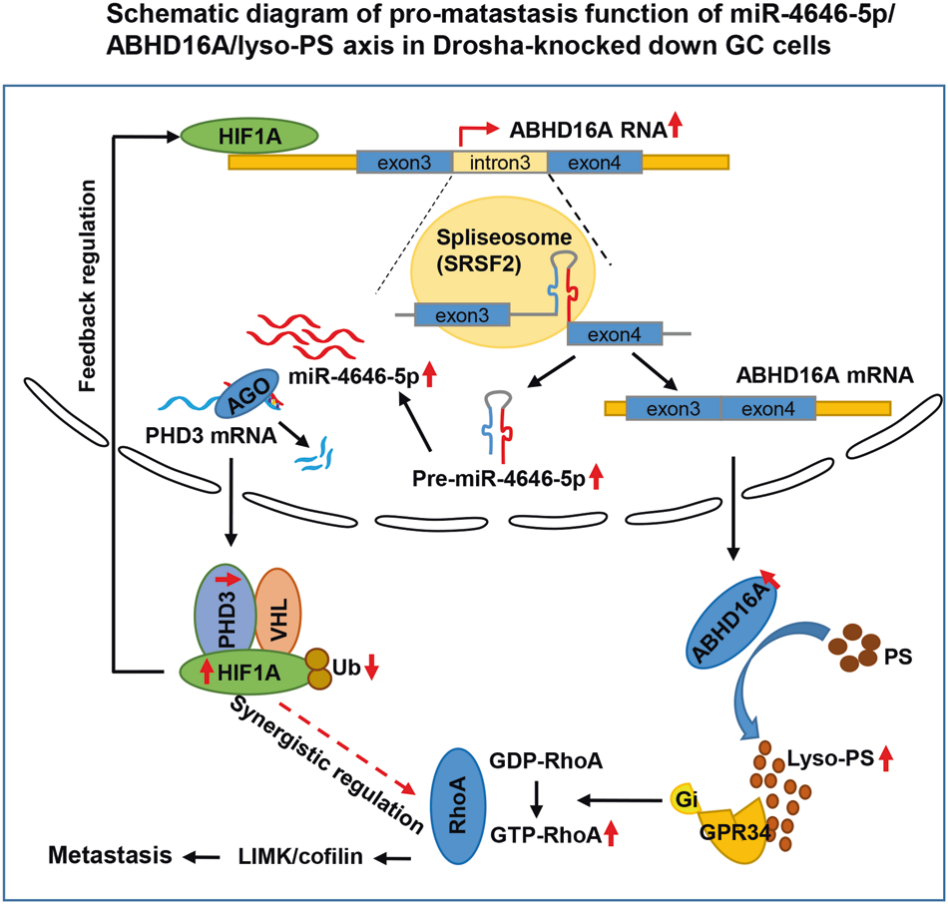
Schematic diagram of pro-metastatic function of miR-4646-5p/ABHD16A/lyso-PS axis in Drosha-low expressed gastric tumor. In Drosha-low expressed gastric cancer cells, mirtronic miR-4646-5p was abnormally up-regulated. The Elevated mirtronic miR-4646-5p is a specific splicing product of intron-3 of the host gene *Abhd16a* with the aid of enhanced SRSF2. The enhanced miR-4646-5p can mitigate ubiquitination of HIF1A by inhibiting the target gene *PHD3*, thus lead to an increased HIF1A. As a transcription factor, HIF1A feedback promotes the expressions of the host gene *Abhd16a* and miR-4646-5p itself. The *Abhd16a* encodes a lipase enzyme ABHD16A, which causes accumulation of lipid metabolite lyso-PS, and then activates RhoA through GPR34/Gi. As synergistic regulation, miRNA-4646-5p can also up-regulate RhoA expression through transcription factor HIF1A. The activation and up-regulation of RhoA synergistically promote gastric cancer metastasis through LIMK/cofilin signaling.

To further confirm miR-4646-5p/ABHD16A-associated pathways in clinic GC tumors, we first conducted gene set enrichment analysis (GESA) using the GC data from TCGA. GSEA enrichment plots showed that miR-4646-5p and ABHD16A expression was positively correlated with Rho-GTPase and cytoskeleton related signaling pathways (Supplementary Fig. S8A). Next, using the TCGA database, GPR34 and RhoA, two of key factors in the miR-4646-5p/ABHD16A/lyso-PS/GPR34/RhoA/LIMK/cofilin signaling, were elevated in GC tumors with metastasis (Supplementary Fig. S8B), which were further confirmed in our cohort of GC patient with metastases (Fig. 7D). In addition, the upregulated ABHD16A, HIF1A, RhoA, and GPR34, and increased phosphorylated LIMK and cofilin were observed in human metastatic gastric tumors using immunohistochemistry (Fig. 7E). Thus, as shown in Fig. 7F, RhoA triggered LIMK/cofilin signaling to promote GC metastasis is closely related with miR-4646-5p/PHD3/HIF1A regulated high expression of RhoA and lyso-PS/GPR34 depended activation of RhoA in GC cells. And the schematic diagram of pro-metastatic function of miR-4646-5p/ABHD16A/lyso-PS axis in Drosha-low expressed gastric tumor was shown in Fig. 8.

## Discussion

Most patients with gastric cancer die from metastasis, but little is known about metastatic mechanisms of GC [33]. In this study, we unravel miR-4646-5p, a nonclassical miRNA, mediated lipid metabolic abnormalities act a pivotal role in gastric cancer metastasis. MiR-4646-5p, a Drosha-independent mirtron, is high expressed in Drosha-decreased gastric cancer and closely correlates with poor survival of GC patients. Unlike the traditional miRNA, whose biogenesis is dependent on Drosha, mirtronic miR-4646-5p is a specific splicing product of intron-3 of the host gene *Abhd16a* under aid of SRSF2. Interestingly, the miRNA-4646-5p can feedback up-regulate its host gene *Abhd16a* expression via PHD3/HIF1A axis. The *Abhd16a* encodes a lipase enzyme ABHD16A, which causes accumulation of lipid metabolite lyso-PS, and then activates RhoA through GPR34/Gi. As a coordinated regulator, miRNA-4646-5p can also regulate *RhoA* expression through transcription factor HIF1A. The activation and up-regulation of RhoA synergistically promote gastric cancer metastasis through LIMK/cofilin signaling.

Mirtron is the most common nonclassical Drosha-independent miRNA, presenting in Drosha reduced GC cells and Drosha low expressed GC tissues. Drosha is critical for classical miRNA biogenesis, and its depletion usually causes significant global miRNA downregulation, participating in tumor progression, such as in breast cancer and non-small cell lung cancer [34, 35]. Herein, we interestingly found a set of nonclassical mirtrons including miR-4646-5p are abnormally elevated in Drosha low expressed gastric tumors and involved in the process, particularly in metastasis, of gastric cancer. We previously found that elevated mirtronic miR-6778-5p strengthens gastric cancer stem cell stemness [12]. Similarly, other reports have documented mirtronic miRNA-1224 is a specific modulator of angiogenesis [36]. And mirtronic miRNA-708 regulates quiescence and self-renewal by antagonizing cell migration and invasion [37]. Consistent with these studies, our findings disclose that nonclassical mirtronic miR-4646-5p plays an essential role in GC poor prognosis by regulating tumor cell metastasis.

Mirtron is derived from the specific splicing of the intron of its host gene [38], but the factors responsible for mirtronic intron splicing are rarely mentioned. It was early observed in patients with myelodysplastic syndrome that SF3B1 and SRFS2 mutations are associated with a downregulated mirtronic miR-3605-5p and miR-4728-5p [39]. A recently study revealed that evaluated SRSF1 and SRSF2 in HCT116 cells significantly increased mirtronic miR-1229-3p and miR-1227-3p expression, implicating that splicing factor could act as a positive regulator for particular species of mirtrons [11]. Likewise, our finding showed that SRSF2 expression was correlated with miR-4646-5p. Silencing SRSF2 in Drosha-knocked down GC cells significantly decreased miR-4646-5p levels, providing further evidence for the participation of splicing machinery in biogenesis of mirtron.

In addition, mirtron is a special type of intronic miRNAs, which could work as partner or antagonist of its host gene by targeting gene functionally associated with its host [40]. Bioinformatics studies predicted that ~20% of intronic miRNAs target their host mRNA transcripts in a feedback loop [41]. For example, hsa-miR-579 was reported to inhibit the transcription of its host ZRF by binding to its 3′-UTR [42]. Besides direct regulation, miRNA also can regulate its host gene function through its targets. For example, miR-641 is not predicted to target its host gene *AKT2*, while its predicted targets, such as PIK3R3, NFAT5, MAPKAP1 and PDK2 are in functional synergy with its host [43]. Here, we found a positive feedback regulation in mirtron and its host, namely miR-4646-5p targets PHD3 to stabilize HIF1A, which positively regulates the expression of host gene *Abhd16a* and miR-4646-5p itself, similar to the feedback loop of mirtronic miR-6778-5p and its host gene *SHMT1* in our previous study [12].

Furthermore, we revealed that miR-4646-5p regulated ABHD16A works as a lipase involved in lipid metabolism. ABHD16A was found as a novel phosphatidylserine-specific lipase. [19]. In the current limited study, the researchers found that ABHD16A might regulate lipid metabolism and bioactive metabolite levels in immune cells, such as dynamically regulating lyso-PS in mouse macrophages [16] and catalyzing PG-G hydrolysis in neutrophils [17]. Intriguingly, in gastric cancer, we confirmed that ABHD16A involves in tumor lipid metabolism, which causes metabolite lyso-PS accumulation to promote cancer metastasis. Additionally, high expression of ABHD16A and metabolite lyso-PS were found in GC tumors tissue with metastasis. Consistent with this finding, lyso-PS was recently shown significant increase in colon cancer tissues and the ascites of gastric cancer patients [21, 22], indicating that ABHD16A and lyso-PS have important biological significance and potential clinical diagnostic value in tumor progression.

More importantly, lyso-PS has emerged as a potent signaling lipid in mammalian physiology [44]. Lyso-PS was reported to induce a variety of cellular responses via its interaction with specific receptors [29]. For example, lyso-PS can provoke eosinophil degranulation via P2Y10 [45], suppress IL-2 production by activated T cells via GPR174 [46], and stimulate chemotactic migration and invasion of colorectal cancer cells via GPR34 [20]. Similarly, our data showed that lyso-PS can activate RhoA activity via GPR34/Gi axis, which proposes a potential new molecular mechanism of lyso-PS on tumor metastasis. In addition, RhoA was reported to be a direct target of HIF1A in breast cancer cells [31]. We found that miR/PHD3-mediated HIF1A stabilization can synergistically promote RhoA expression. Meanwhile, RhoA is a metastasis-related molecule, which promotes cell migration and invasion by regulating phosphorylation of LIMK/cofilin [47]. Indeed, our works prove that RhoA activation and upregulation trigger LIMK/cofilin signaling, providing a reliable clue for gastric cancer cell metastasis.

In general, our research highlights the feedback regulation between mirtronic miR-4646-5p and its host gene *Abhd16a*, and their function in lipid metabolism and metastasis of gastric cancer. Namely, miR-4646-5p feedback promotes *Abhd16a* mRNA transcription and expression of ABHD16A protein, involving in lipid metabolism to cause lyso-PS accumulation, thus stimulates activation of GPR34 receptor to trigger RhoA/LIMK/cofilin signaling in fueling gastric cancer metastasis. Our findings open new insights for gastric cancer metastasis.

## Supplementary information

Supplementary Table S1

Supplementary Table S2

Supplementary Table S3

Supplementary Table S4

Supplementary Figure 1

Supplementary Figure 2

Supplementary Figure 3

Supplementary Figure 4

Supplementary Figure 5

Supplementary Figure 6

Supplementary Figure 7

Supplementary Figure 8

Supplementary Figure legends
