# Supplementary material for: Mirtronic miR-4646-5p promotes gastric cancer metastasis by regulating ABHD16A and metabolite lysophosphatidylserines
Source: Cell Death Differ. 2021 Apr 19;28(9):2708–27. doi: 10.1038/s41418-021-00779-y (PMC8408170; doi:10.1038/s41418-021-00779-y)
Supplement: Supplementary file 1 — Supplementary Table S1 [file 41418_2021_779_MOESM1_ESM.docx]

| **Supplemental Table 1. Core sequences of shRNA against target genes** | |
| --- | --- |
| **Target gene** | **shRNA sequences** |
| **NC** | 5’-TTCTCCGAACGTGTCACGT-3’ |
| ***Drosha*** | 1# 5’-CGAGUAGGCUUCGUGACUUTT-3’  2# 5’-ACGAAGCTCGATGAAGATTTA-3’ |
| ***PHD3*** | 1# 5’-GTGGCTTGCTATCCGGGAAAT-3’  2# 5’-CACCTGCATCTACTATCTGA-3’ |
| ***Abhd16a*** | 1# 5’-GCCUCAAUUUAUAGCCGAUTT-3’  2# 5’-AUCUGUAAAAGUCAUUCUGAA-3’ |
| ***GPR34*** | 1# 5’-CAGTTTGGATCGCTATATA-3’  2# 5’-UAAUAUGAUACAUUAUUCGGA-3’ |
| ***SRSF2*** | 1# 5’-UCGAAGUCUCGGUCCCGCACUCG-3’  2# 5’-GAGGACGCUAUGGAUGCCAUGGACG -3’ |
