# Supplementary material for: Mirtronic miR-4646-5p promotes gastric cancer metastasis by regulating ABHD16A and metabolite lysophosphatidylserines
Source: Cell Death Differ. 2021 Apr 19;28(9):2708–27. doi: 10.1038/s41418-021-00779-y (PMC8408170; doi:10.1038/s41418-021-00779-y)
Supplement: Supplementary file 2 — Supplementary Table S2 [file 41418_2021_779_MOESM2_ESM.docx]

| **Supplementary Table 2. Primers used for *Abhd16a* genes amplification, 5’ and 3’ splicing sites mutagenesis** | |
| --- | --- |
| **Gene name** | **Primer** |
| ***Abhd16a*** | **FW 5’-CTTCAGTATTCTGGTCCATCTCT-3’**  **REV 5’-CACACCTGCCAGAAGTAGC-3’** |
| **5’splice site mutation** | **FW 5’-GTCAGTGTGGTTTCAAAGGT-3’**  **REV 5’-GTGGGAAGGGAGAGGGACAAT-3’** |
| **3’ splice site mutation** | **FW 5’-CTCAGTGTGGTTTCAAAGGTGR-3’**  **REV 5’- CTGGGAAGGGAGAGGGACAATG-3’** |
