# Supplementary material for: Mirtronic miR-4646-5p promotes gastric cancer metastasis by regulating ABHD16A and metabolite lysophosphatidylserines
Source: Cell Death Differ. 2021 Apr 19;28(9):2708–27. doi: 10.1038/s41418-021-00779-y (PMC8408170; doi:10.1038/s41418-021-00779-y)
Supplement: Supplementary file 3 — Supplementary Table S3 [file 41418_2021_779_MOESM3_ESM.docx]

| **Supplemental Table 3. Primer sequences used for qRT-PCR analysis** | | |
| --- | --- | --- |
| Gene | Primer sequence |  |
| *Abhd16a* | F: CCCCCGGCTCTACAAAATCTAC |  |
|  | R: GATAGTACGTATCCCAGGAGCTG |  |
| *SRSF1* | F:TGCCTACATCCGGGTTAAAG |  |
|  | R:CTGCTGTTGCTTCTGCTACG |  |
| *SRSF2* | F: CGACCTCCAAGTCCAGATCCG |  |
|  | R: GGGACTCTTCGATCGCGACCT |  |
| *SRSF5* | F: GAGGCTTTGGTTTTGTGGAA |  |
|  | R: CGAGCCCTAGCATGTTCAAT |  |
| *β-actin* | F: CTGGAAGGTGGACAGCGAGG |  |
|  | R: CTGGAAGGTGGACAGCGAGG |  |
| *PHD3* | F: ATACTACGTCAAGGAGAGGT |  |
|  | R: TCAGCATCAAAGTACCAGA |  |
| *GPR34* | F: GGGACTGGTTGGGAACATAA |  |
|  | R: GAAAGGGAGGCAGAAGATGA |  |
| *P2Y10* | F: GCAGGATTTGTGATCCCAGT |  |
|  | R: CCTCTCACTGATCCCTTGGA |  |
| *GPR174* | F: TTCCTTGCCACTGAGGATCT |  |
|  | R: AAAATCGTCGCACACTGATG |  |
| *GAPDH* | F: CTTTGGTATCGTGGAAGGACTC |  |
|  | R: GTAGAGGCAGGGATGATGTTCT |  |
| *RhoA* | F: TTCCATCGACAGCCCTGATAGTTTA |  |
|  | R: CACGTTGGGACAGAAATGCTTG |  |
| hsa-miR-4646-5p | F: GCGACTGGGAAGAGGAGCT |  |
|  | R: AGTGCAGGGTCCGAGGTATT |  |
|  | RT:GTCGTATCCAGTGCAGGGTCCGAG  GTATTCGCACTGGATACGACTCCCTC |  |
| U6 | F: CTCGCTTCGGCAGCACA |  |
|  | R: AACGCTTCACGAATTTGCGT |  |
