# Supplementary material for: Mirtronic miR-4646-5p promotes gastric cancer metastasis by regulating ABHD16A and metabolite lysophosphatidylserines
Source: Cell Death Differ. 2021 Apr 19;28(9):2708–27. doi: 10.1038/s41418-021-00779-y (PMC8408170; doi:10.1038/s41418-021-00779-y)
Supplement: Supplementary file 4 — Supplementary Table S4 [file 41418_2021_779_MOESM4_ESM.docx]

| **Supplemental Table 4. Primer sequences used for PCR analysis in ChIP assay** | |
| --- | --- |
| **Gene** | **Primer sequences** |
| ***Abhd16a*** | **F: 5’-TCTCTTTCTTCTCCACTTGCT-3’**  **R: 5’-GCTGAGTGAAAATAGAAAGCCACA-3’** |
| ***RhoA*** | **F: 5’-CTCCTGAGCAATAGTGGATGAGC-3’**  **R: 5’-GACGTGCGCGCCTCCGAGT-3’** |
