# Supplementary figures and images for: Mirtronic miR-4646-5p promotes gastric cancer metastasis by regulating ABHD16A and metabolite lysophosphatidylserines
Source: Cell Death Differ. 2021 Apr 19;28(9):2708–27. doi: 10.1038/s41418-021-00779-y (PMC8408170; doi:10.1038/s41418-021-00779-y)

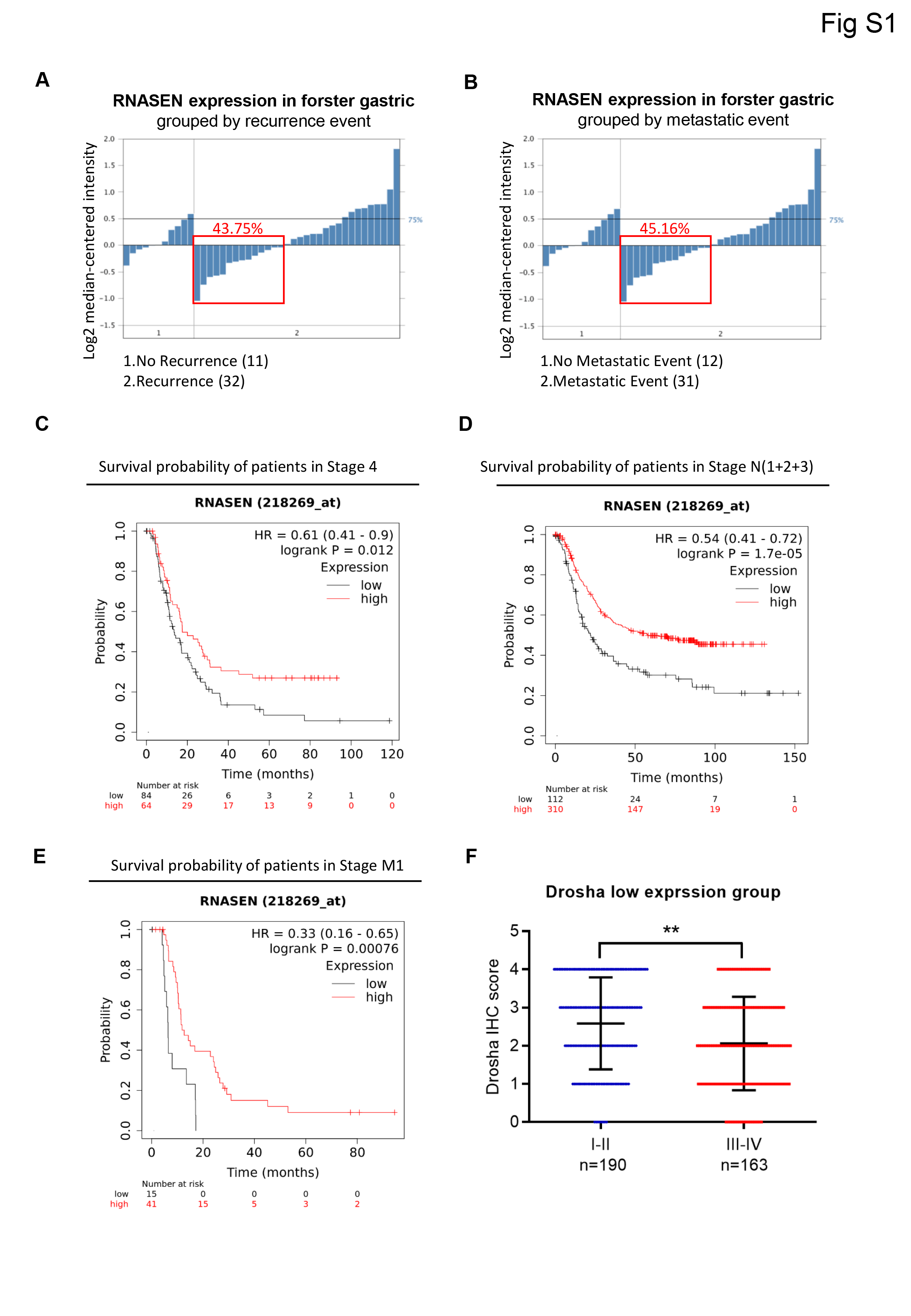

Supplement: Supplementary file 5 — Supplementary Figure 1 [file 41418_2021_779_MOESM5_ESM.tif]

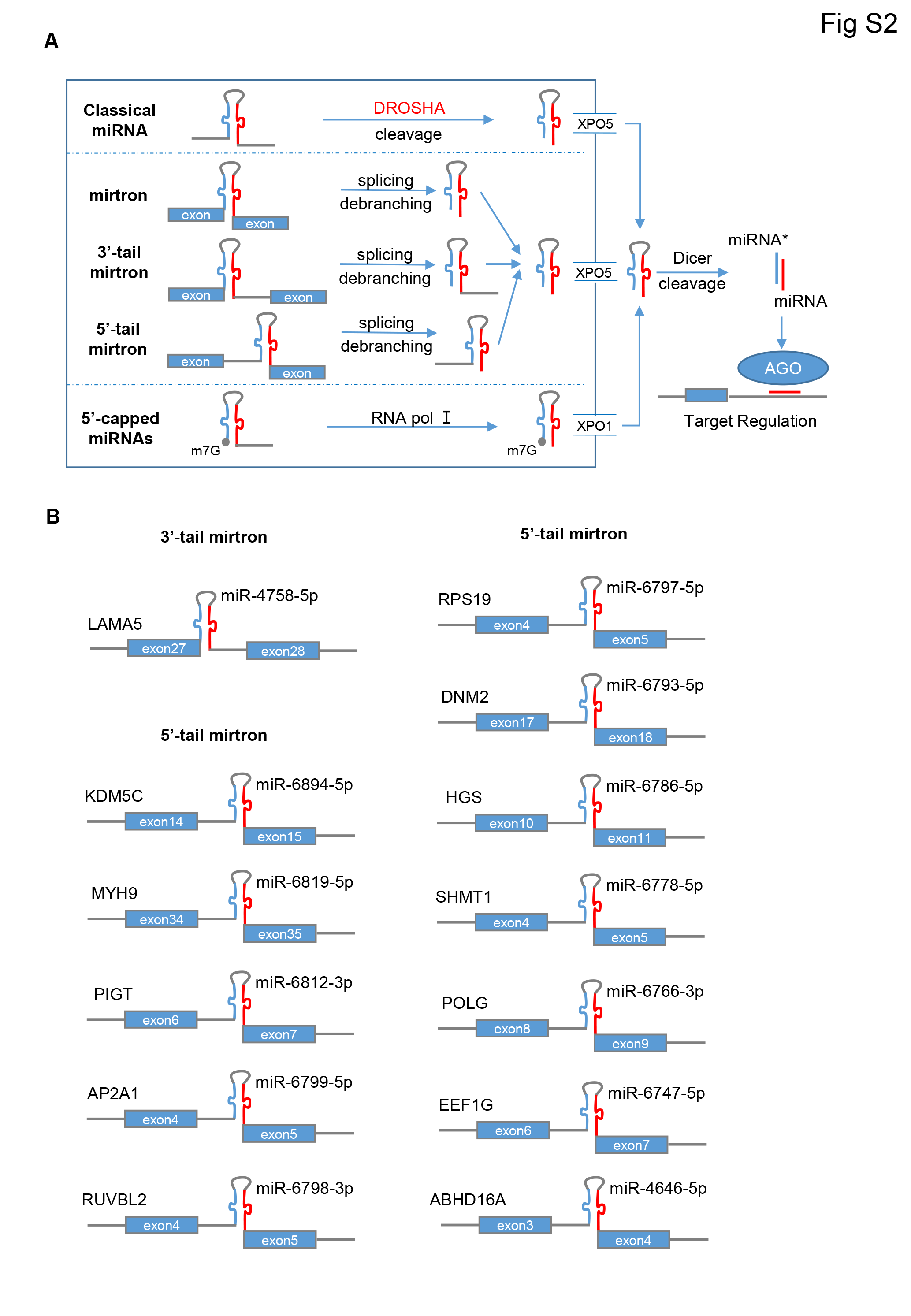

Supplement: Supplementary file 6 — Supplementary Figure 2 [file 41418_2021_779_MOESM6_ESM.tif]

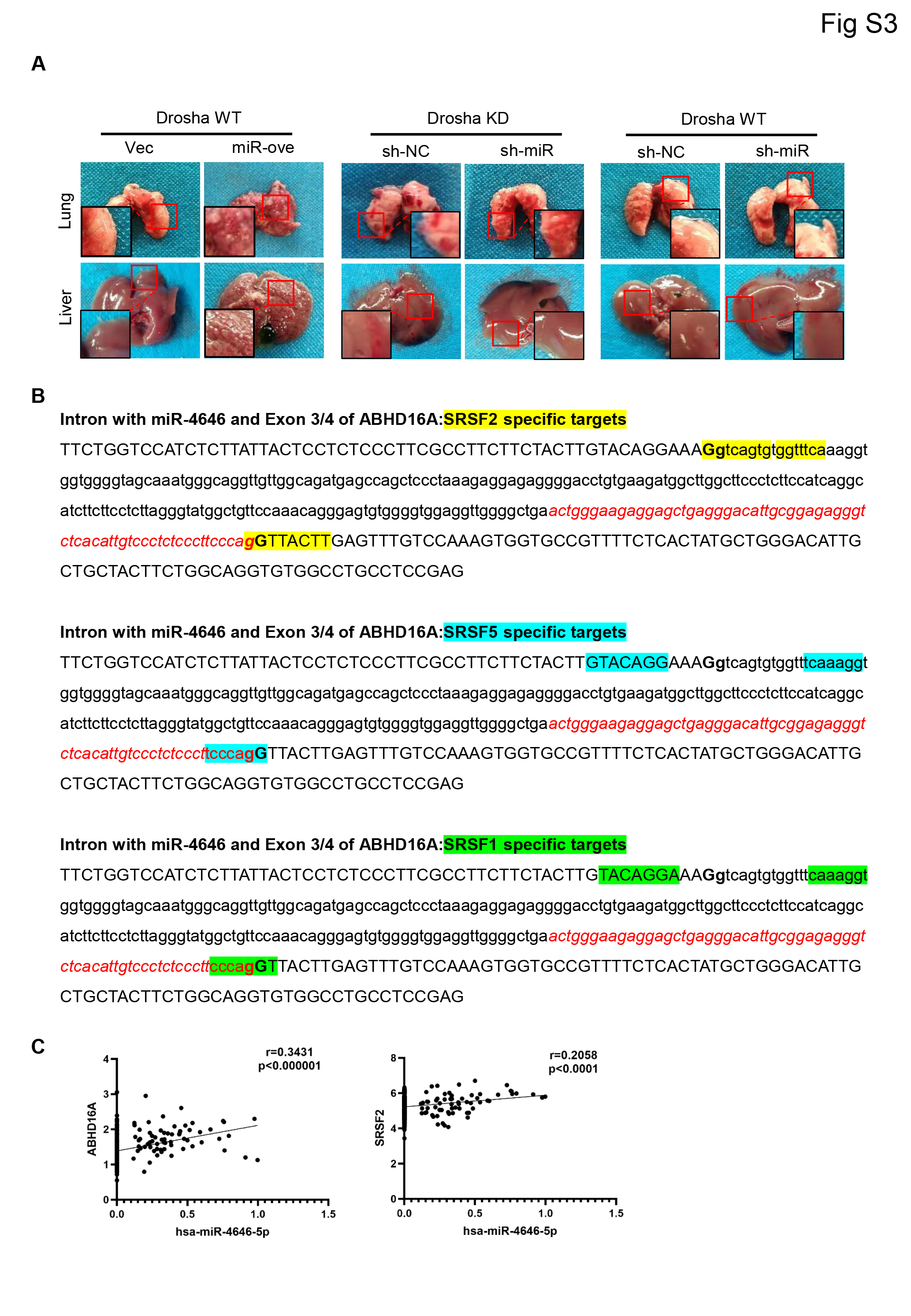

Supplement: Supplementary file 7 — Supplementary Figure 3 [file 41418_2021_779_MOESM7_ESM.tif]

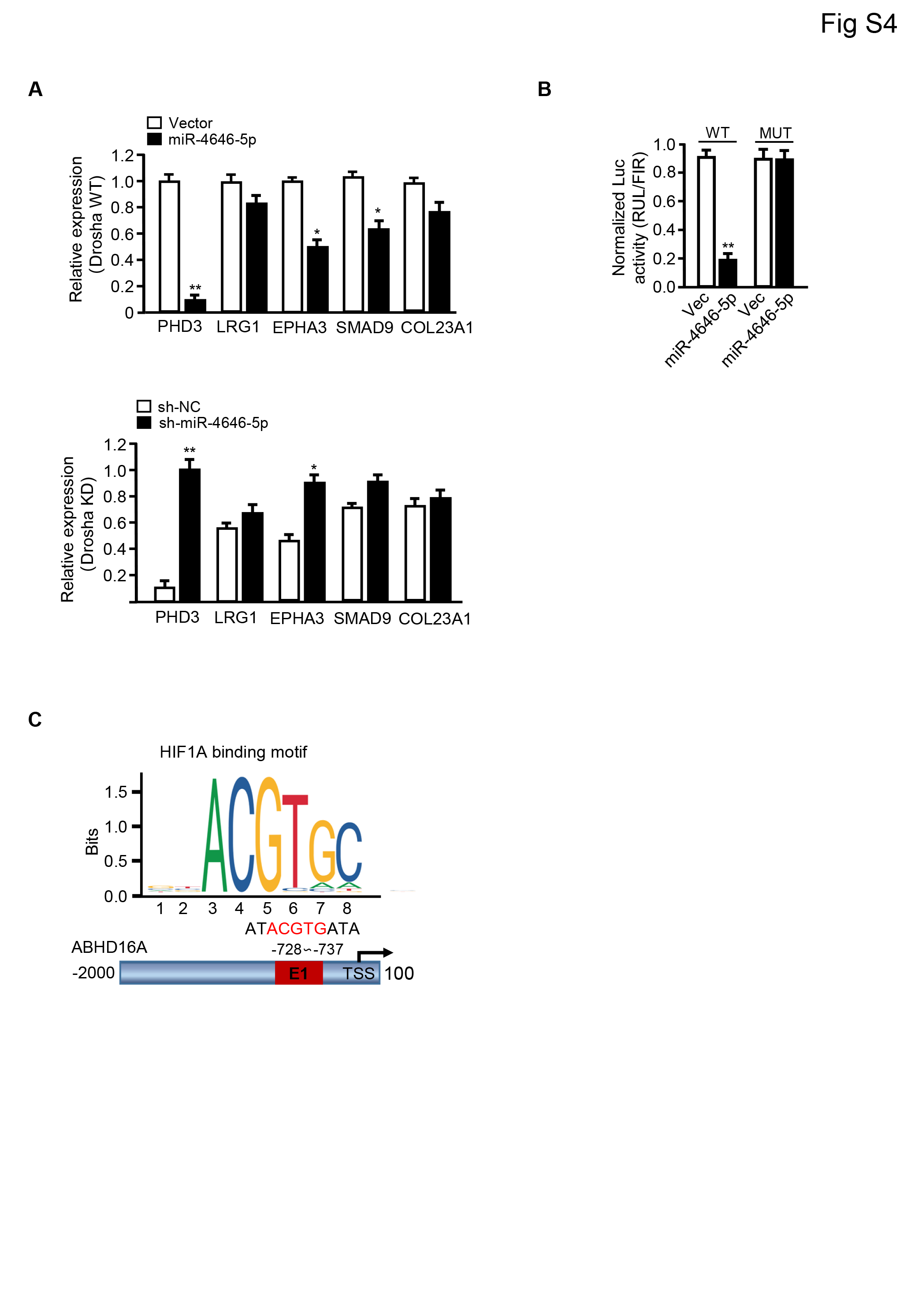

Supplement: Supplementary file 8 — Supplementary Figure 4 [file 41418_2021_779_MOESM8_ESM.tif]

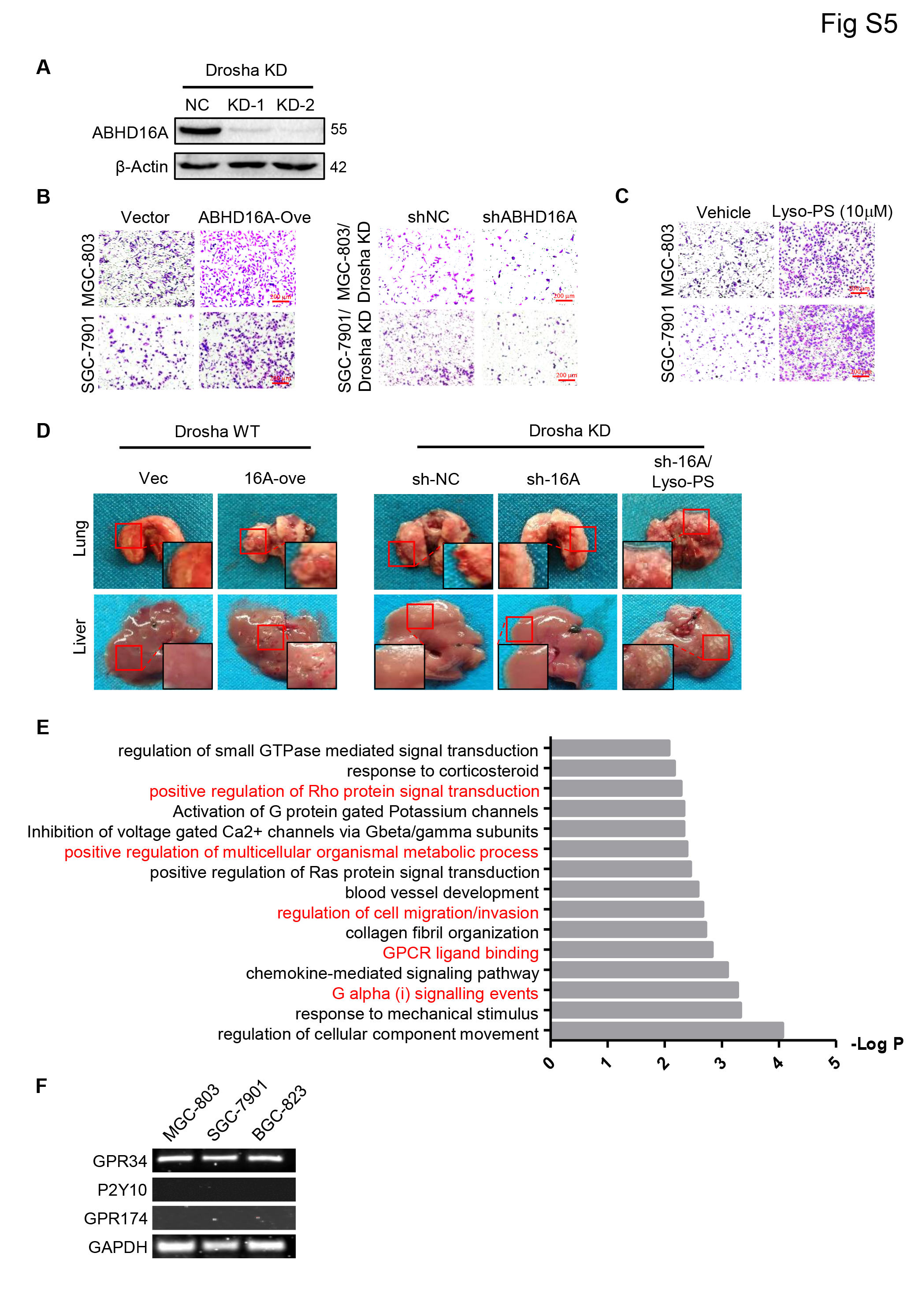

Supplement: Supplementary file 9 — Supplementary Figure 5 [file 41418_2021_779_MOESM9_ESM.tif]

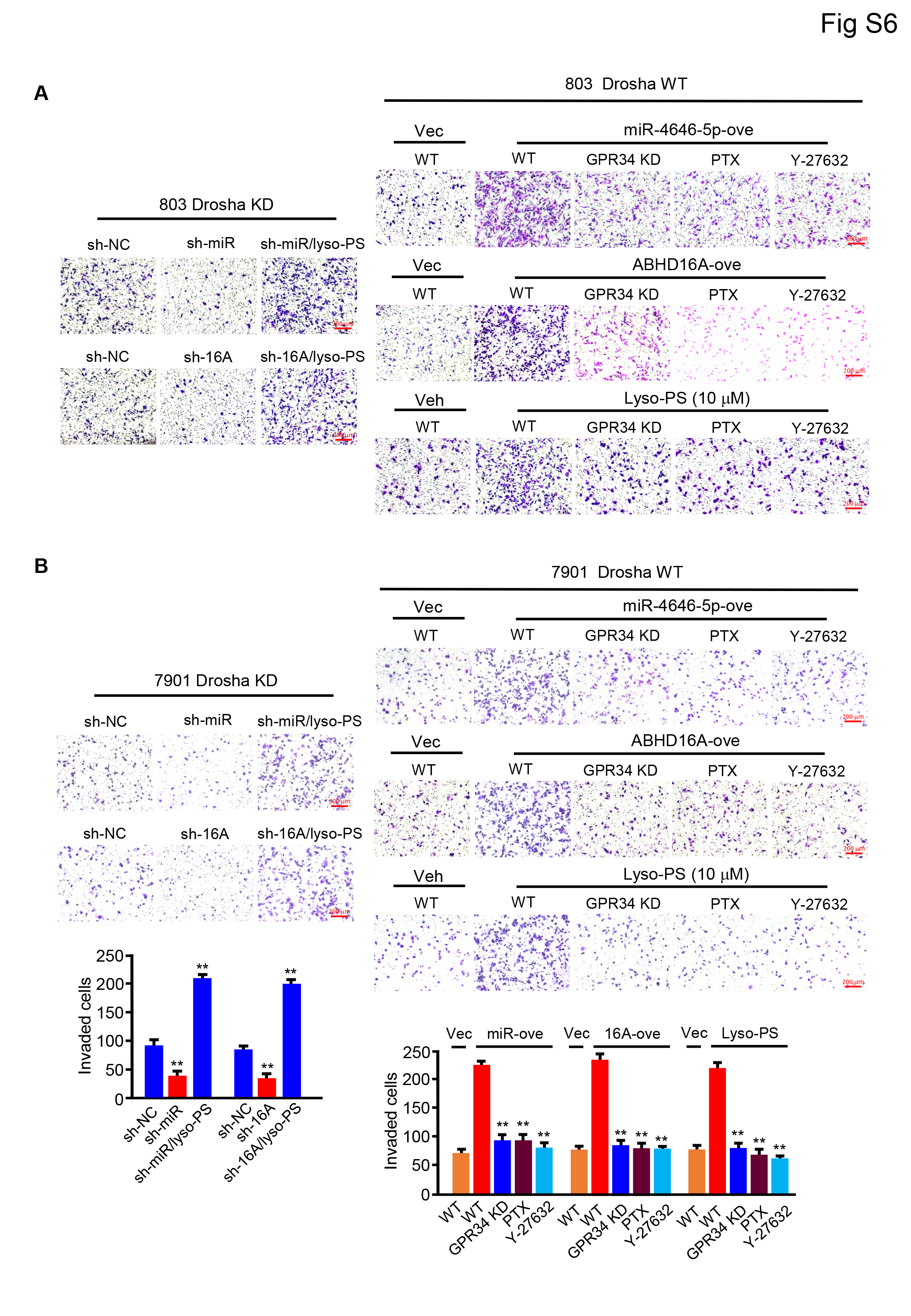

Supplement: Supplementary file 10 — Supplementary Figure 6 [file 41418_2021_779_MOESM10_ESM.tif]

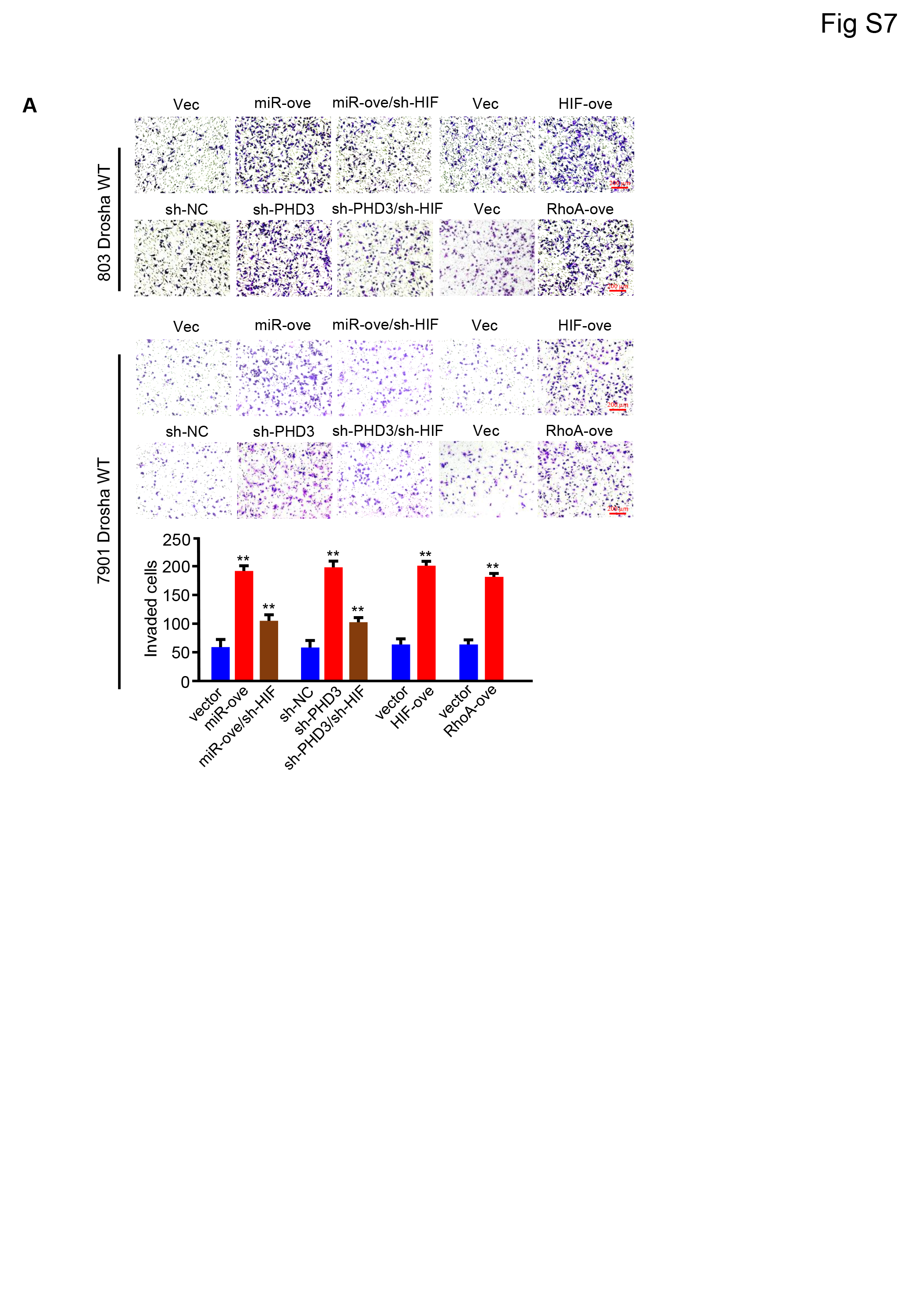

Supplement: Supplementary file 11 — Supplementary Figure 7 [file 41418_2021_779_MOESM11_ESM.tif]

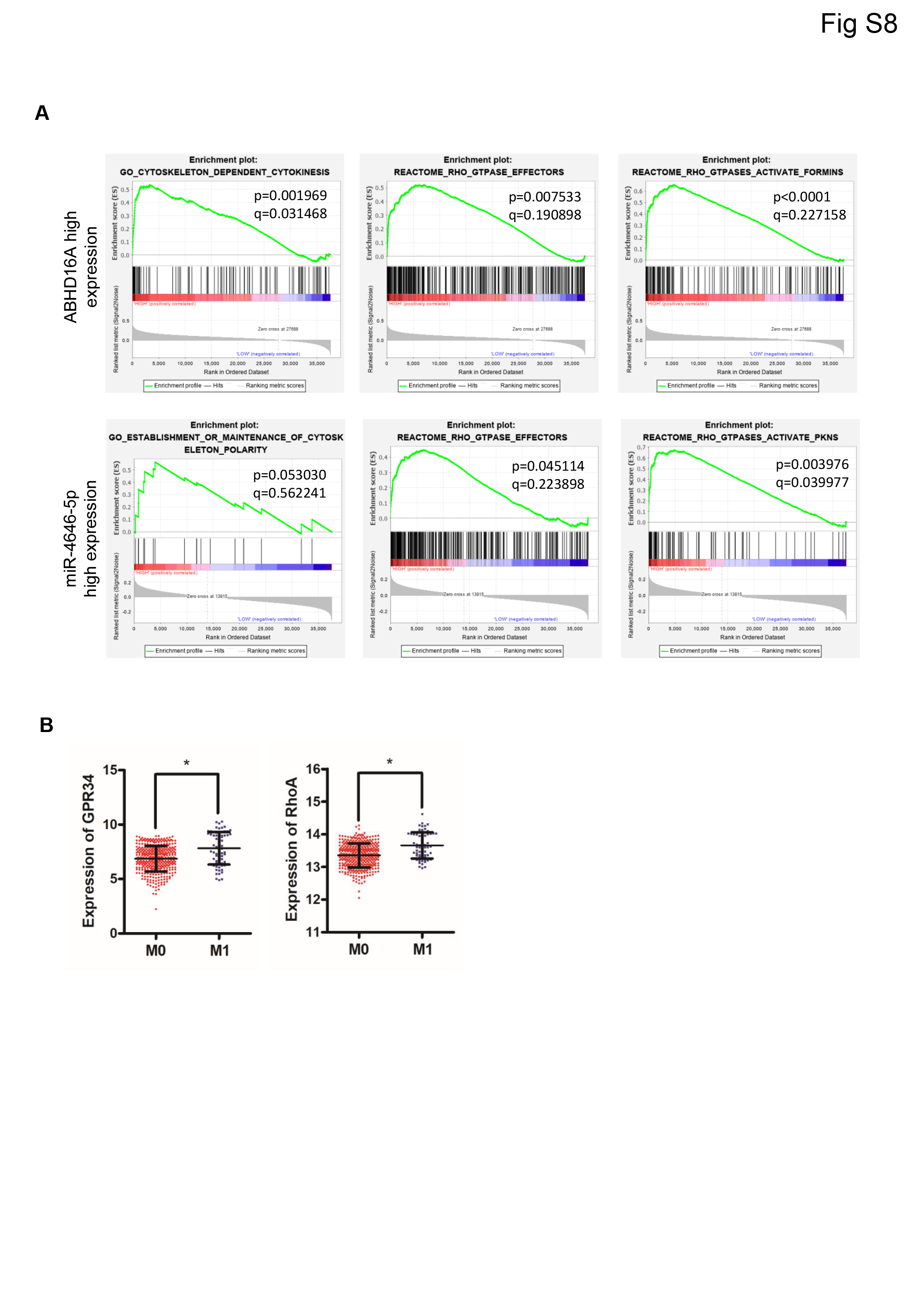

Supplement: Supplementary file 12 — Supplementary Figure 8 [file 41418_2021_779_MOESM12_ESM.tif]
