# Supplementary material for: Mirtronic miR-4646-5p promotes gastric cancer metastasis by regulating ABHD16A and metabolite lysophosphatidylserines
Source: Cell Death Differ. 2021 Apr 19;28(9):2708–27. doi: 10.1038/s41418-021-00779-y (PMC8408170; doi:10.1038/s41418-021-00779-y)
Supplement: Supplementary file 13 — Supplementary Figure legends [file 41418_2021_779_MOESM13_ESM.docx]

**Supplementary Figure legends**

**Figure S1. Patients with reduced Drosha still maintained high malignant characteristics and had a worse prognosis.**

(A, B). Oncomine data showing RNASEN (Drosha) expression in recurrent GC patients (n=32) and non-recurrent GC patients (n=11) (A), or in GC patients with (n=31) or without metastasis (n=12) (B). Among them, 43.75% patients with recurrence (n=14) (A) or 45.16% patients with metastasis (n=14) (B) had low expressed Drosha in GC tumors. (C-E). GC patients with low Drosha expression had a poor prognosis. Kaplan-Meier (KM) survival curve for GC patients in clinical stage 4 (n=148) (C), had lymph node metastatic event (N1+2+3, n=422) (D) or distant metastasis (M1, n=56) (E) (data information from <http://kmplot.com/>). (F). Low IHC staining scores (IHC<6) of Drosha proteins in different clinical stages. 46.17% GC patients (n=163) with Drosha low expression were in Stage Ⅲ-Ⅳ.

**Figure S2. Non-classical miRNAs biogenesis and Drosha-independent mirtrons.**

(A). Schematic diagram of classical and non-classical microRNA (miRNA) biogenesis. Mirtrons including 3′-tail mirtron, 5′-tail mirtron, and 5′-capped miRNAs belong to Drosha-independent miRNAs (non-classical microRNAs). (B). Minigene structures of some aberrant elevated mirtrons identified by microarray in Drosha-knockdown MGC-803 cells. The precursor of mirtrons and its host genes were analyzed using UCSC database (http://genome.ucsc.edu/).

**Figure S3. Mirtronic miR-4646-5p is a specific splicing product of intron-3 of the host gene *Abhd16a* under the aid of SRSF2.**

(A) Representative images of lung and liver metastases in the subgroups. (B). The position of splicing signals predicted by online bioinformatics tool of Human Splicing Finder. SRSF2-specific targets are highlighted in yellow, SRSF5-specific targets in blue, SRSF1-specific targets in green. Sequences of mirtron were labeled using italic red characters, and introns labeled using lowercase, exons labeled using uppercase. **(**C). miR-4646-5p expression in GC patients correlated with the expression of ABHD16A (left panel, p<0.000001) and SRSF2 (right panel, p<0.0001). The expression data were downloaded from UCSC Xena.

**Figure S4.** **miR-4646-5p targets PHD3 to stabilize HIF1A to feedback up-regulate the host gene *Abhd16a* expression.**

(A). qRT-PCR to test PHD3, LRG1, EPHA3, SMAD9 and COL23A1 expression in MGC-803 cells transfected with ectopic miR-4646-5p or control vector (upper panel), and MGC-803 cells with Drosha-knockdown or Drosha and miR-4646-5p double knockdown (down panel). (B). The effect of ectopic miR-4646-5p on luciferase activities was tested in HEK-293T cells co-transfected with either the WT reporter or the MUT reporter (right panel). (C). HIF1A was predicted to be a potential transcription factor of ABHD16A using JASPAR database (http://jaspar.genereg.net/). The HIF1A binding motif in ABHD16A prompter is shown.

**Figure S5. ABHD16A caused accumulation of lipid metabolite lyso-PS results in GC metastasis.**

(A). shRNA-mediated knockdown efficiencies of ABHD16A in Drosha-silenced MGC-803 cells were verified by western blotting. (B). Transwell was used to test cell invasion ability for MGC-803 and SGC-7901 cells with ectopic ABHD16A or control vector (left panels), or for Drosha and ABHD16A doubly knocked down MGC-803 and SGC-7901 cells and its controls (right panels). (C). Transwell assay to assess cell invasion ability for MGC-803 and SGC-7901 treated with or without lyso-PS (10 μM). (D) Representative images of lung and liver metastases in the subgroups. (E). The significantly enriched KEGG pathways in MGC-803 treated with or without lyso-PS analyzed by KOBAS (<http://kobas.cbi.pku.edu.cn/>). (F). RT-PCR analysis of lyso-PS receptors (GPR34, P2Y10 and GPR174) expression in gastric cancer cells, GAPDH is the internal control.

**Figure S6. Lyso-PS activated RhoA via GPR34 receptor promotes GC metastasis.**

(A). Cell invasion abilities of indicated MGC-803 cells were assessed by Transwell assay. Left panels show the invaded cells of Drosha knockdown MGC-803 cells with miR-4646-5p/ABHD16A silencing or miR-4646-5p/ABHD16A silencing with lyso-PS stimulation. Right panels show the invaded cells of MGC-803 cells with wild type GPR34, shGPR34, or GC cells treated with PTX (inhibitor of Gα_i_, 100 ng/ml) or Y-27632 (RhoA inhibitor, 10 μM) under miR-4646-5p or ABHD16A overexpression or lyso-PS treatment. (B). Cell invasion abilities of SGC-7901 cells grouped as above were assessed by Transwell assay. The histograms show the average invaded cells each view (**p<0.01).

**Figure S7. miR-4646-5p and ABHD16A play a role in regulating RhoA expression and RhoA activation in vivo and in vitro.**

(A). Cell invasion abilities of indicated MGC-803 and SGC-7901 cells were assessed by Transwell assay. The invaded cells of GC cells with ectopic miR-4646-5p, HIF1A and RhoA or silenced PHD3 and the controls, or HIF1A reduced MGC-803 cells with ectopic miR-4646-5p or silenced PHD3. The histograms show the average invaded cells each view (**p<0.01).

**Figure S8. miR-4646-5p/ABHD16A/Lyso-PS stimulated activation of RhoA trigger LIMK/cofilin signaling to promote GC metastasis in clinic.**

(A). Gene‐set enrichment analysis (GSEA) of ABHD16A and miR-4646-5p using TCGA data. (B). Expression of GPR34 (left panel) or RhoA (middle panel) in gastric tumor patients with distant metastases (M1) and non-distant metastases (M0). The expression data and patient information were downloaded from UCSC Xena (**p<0.05).
